# Theoretical treatment of tension transients in muscle following sudden changes in orthophosphate concentration: implications for energy transduction

**DOI:** 10.1007/s10974-025-09698-8

**Published:** 2025-07-14

**Authors:** Alf Månsson

**Affiliations:** Department of Chemistry and Biomedical Sciences, Faculty of Health and Life Science, Linnaeus University, 391 82 Kalmar, Sweden

**Keywords:** Inorganic phosphate, Myosin force generation, Phosphate transient, Kinetic scheme, Mechanokinetic model, Multistep phosphate release

## Abstract

The relative timing of the force-generating power stroke and release of the ATP-hydrolysis product orthophosphate (Pi) in actomyosin energy transduction is debated. It may be explored by studying the tension response to sudden changes in [Pi] during isometric muscle contraction (Pi-transients; rate constant k_Pi_) and by the rate of redevelopment of isometric force (k_tr_) after a period of unloaded shortening at varied [Pi]. Most studies of these types are interpreted using simple kinetic schemes that ignore the range of elastic strains of actin-attached myosin cross-bridges. We found that the only simple scheme which accounts for the experimental findings of single exponential Pi-transients with k_Pi_ ≈ k_tr_ has force-generation coincident with actin-myosin attachment. This characteristics could compromise the high power output of muscle. We therefore turned to a mechanokinetic model, allowing consideration of the varying elastic cross-bridge strains. Our model assumes Pi-release between cross-bridge attachment and the force-generating power stroke. However, power strokes only occur if cross-bridges attach in a pre-power-stroke state with zero or negative elastic strain (counteracting shortening). The model suggests two components of the Pi-transients. One is attributed to slow cross-bridge detachment from the pre-power-stroke state at positive elastic strain upon Pi-binding. The other is due to Pi-induced shifts in equilibrium with rapid power stroke reversal. The slow component dominates for all parameter values tested but the fast component is ubiquitous, predicting a biphasic Pi-transient in disagreement with experiments. Strikingly, however, the mechanokinetic model gives different predictions than apparently similar simple kinetic schemes and we do not rule out the existence of parameter values leading to a negligible fast component. We also show that the assumption of secondary Pi-binding sites on myosin outside the active site removes the fast component albeit without predicting that k_tr_ ≈ k_Pi_. Additional studies are required to finally corroborate that k_tr_ ≈ k_Pi_ in experiments but also to further develop mechanokinetic models combined with multistep Pi-release.

## Introduction

Muscle contraction is the result of cyclic interactions between myosin II motor domains and actin filaments, powered by turnover of adenosine triphosphate (ATP). A key step in chemomechanical energy transduction is the release of inorganic phosphate (orthophosphate; Pi) from the active site of myosin (reviewed in (Robert-Paganin et al. 2020; Rassier and Månsson 2025)). In solution-studies of isolated myosin II and actin, this step is associated with a large drop in free energy related to the main force-generating structural changes in muscle cells. The most important of these changes is a swing of the light-chain-binding domain of myosin, acting as a lever arm (reviewed in (Robert-Paganin et al. 2020; Rassier and Månsson 2025)). This swing, often (as here) denoted the power stroke is associated with structural rearrangements around the myosin active site and closure of a cleft between the upper and lower 50 kD domain of myosin to increase actin affinity. Whereas these processes have been extensively studied, the relative temporal order of the events are debated (reviewed in (Geeves 2016; Robert-Paganin et al. 2020)). By only considering the order between Pi-release from the active site and the lever arm swing there are currently several models, each seemingly supported by a given set of experimental results and model studies (Debold 2021; Månsson et al. 2023). Some studies favour the view that Pi is released from the active site strictly before the power stroke (Smith 2014; Llinas et al. 2015; Rahman et al. 2018; Offer and Ranatunga 2020; Hwang et al. 2021; Moretto et al. 2022). Others favor the opposite view (Kawai and Halvorson 1991; Dantzig et al. 1992; Tesi et al. 2000; Muretta et al. 2015; Woody et al. 2019), i.e. with Pi released after the power stroke, possibly combined with branched pathways (Linari et al. 2010; Debold et al. 2011; Jarvis et al. 2018; Scott et al. 2021; Marang et al. 2025). However, there are also models assuming loose coupling between Pi-release and force-generation so that Pi may be released either before or after the power-stroke (Malnasi-Csizmadia and Kovacs 2010; Caremani et al. 2013; Rohde et al. 2017) with different probability depending on the exact condition. Finally, several recent studies have considered the possibility that Pi binds to secondary sites on myosin after it has left the active site (Smith 2014; Llinas et al. 2015; Moretto et al. 2022).

One type of experimental results that seems to support the idea that Pi is released from the active site after the force-generating structural transition, is the time course of the isometric tension transients (Pi-transients) seen when the myofibrillar Pi-concentration is suddenly changed during isometric contraction. The effect of a sudden increase in [Pi] on isometric tension can be studied in skinned muscle cells by Pi-release from caged Pi inside the myofilament lattice (Millar and Homsher 1990; Dantzig et al. 1992). Effects of both an increase and a decrease in [Pi] can be studied in contracting myofibrils by rapidly switching the surrounding solution (Tesi et al. 2000). The results of experiments of these types suggest (Dantzig et al. 1992; Tesi et al. 2000; Stehle 2017) that the Pi-transient is well fitted by a single exponential function (rate constant k_Pi_) following a brief (< 10ms) delay. Complementary information about the Pi-release and Pi-re-binding mechanisms can be gained by studying the rate of redevelopment of isometric force after a release to zero force and a restretch (Brenner and Eisenberg 1986) at varied [Pi] (Tesi et al. 2000; Stehle 2017, 2024). The rate constant for the rise of force under these conditions (“force redevelopment” below) is denoted k_tr_. This rate has been found to be similar (< 10% faster) to the initial rate of rise of force during isometric contraction of a myofibril at full activation (Tesi et al. 2000). Evidence was recently presented (Stehle 2017) that Pi-transients in response to increased (but not reduced) [Pi] are speeded up by non-uniform sarcomere behavior along a myofibril from the guinea pig cardiac ventricle. This would explain the apparent conundrum in earlier experiments that k_Pi_ > k_tr_ for an increase in [Pi] whereas k_Pi_ ≈ k_tr_ for reduced [Pi] (Millar and Homsher 1990; Tesi et al. 2000). This result and additional findings (Stehle 2017), could mean that generally k_Pi_ ≈ k_tr_ on the half-sarcomere level. This is what we will assume in our analysis while being aware that there may be differences between the results in (Stehle 2017) using cardiac myofibrils and other experimental results usually obtained using skeletal muscle preparations as further discussed below.

Both k_Pi_ and k_tr_ increase from a non-zero value at zero [Pi] either linearly (Stehle 2017) or reaching saturation with a rectangular hyperbolic dependence on [Pi] (Dantzig et al. 1992; Tesi et al. 2000; Stehle 2024). In the latter case, half saturation is generally reached at a [Pi] in the range 1–10 mM. As mentioned above, the effects of varied [Pi] on the Pi-transients have been interpreted to mean that Pi is released from the active site *after* the force-generating structural change. Results from several other types of experimental studies at varied [Pi] accord with this view (Fortune et al. 1991; Kawai and Halvorson 1991; Ranatunga 1999; Ranatunga et al. 2002; Tesi et al. 2002; Woody et al. 2019; Kawai et al. 2021; Scott et al. 2021; Stehle 2024) (reviewed in (Kawai 2025)), Moreover, these studies generally seem to suggest that the force-generating event in the cross-bridge is coincident with or occurs in close association with cross-bridge attachment. Indeed, this seems to be the most straightforward interpretation (as corroborated in our analysis below) when the experimental data are interpreted in terms of simple kinetic schemes (Dantzig et al. 1992; Tesi et al. 2000; Stehle 2024) (however, see (Caremani et al. 2008). In such schemes all cross-bridges in a given biochemical state, e.g. an actin-attached state with both ADP and Pi at the active site would be treated as one state even if the elastic strain varies in the population. Thus, only one transition rate constant to a given neigbhoring biochemical state is considered. One may question if such simplified schemes can be faithfully used to interpret experimental results from steady-state isometric contraction (Månsson et al. 2023). Even though this is a steady-state and static condition one would expect a range of elastic strains for the attached cross-bridges in a real muscle due to ubiquitous thermal fluctuations under physiological conditions. Because elastic energies contribute to the free energy of a state there would be a distribution of force and strain-dependent rate constants (Huxley 1957; Hill 1974; Rassier and Månsson 2025) for transitions into and out of a given biochemical state. Moreover, associated with the differences in rate constants, a given biochemical state may be accessible to certain inter-state transitions only for some cross-bridge strain levels.

The present paper takes the above experimental and theoretical insights as starting points. We first re-consider interpretation of Pi-transients using simple kinetic schemes similar to some of those tested previously (Kawai and Halvorson 1991; Dantzig et al. 1992; Tesi et al. 2000, 2002; Ranatunga et al. 2002; Ranatunga 2010; Kawai et al. 2021; Stehle 2024) (reviews by (Stehle and Tesi 2017; Kawai 2025)). The results raise questions on how effective energy transduction can be accomplished if the power stroke is conincident with the cross-bridge attachment step itself as seems to be the conclusion from analyses using the simple schemes. The possibility exists (as suggested previously (Smith 2014)) that more complex mechanokinetic models that take elastic cross-bridge strain into account could lead to alternative interpretations. We therefore expanded the analysis using kinetic schemes to a mechanokinetic model developed from previous work (Månsson 2016; Moretto et al. 2022). This model could approximately account for the Pi-transients and their Pi-dependence with separate attachment and power stroke transitions and with Pi-release before the power stroke. However, it seems challenging to perfectly reproduce the single exponential behavior with k_tr_ ≈ k_Pi_ as observed in experiments. Overall, our results suggest remaining enigmas in energy transduction related to the timing of Pi-release and force-generation that cannot be fully reproduced by any of the mentioned models. A multistep Pi-release mechanism considered recently (Llinas et al. 2015; Robert-Paganin et al. 2020; Moretto et al. 2022) may amend the situation but more detailed investigations of this possibility will be required.

## Model

### Simple kinetic schemes

**Fig. 1:**
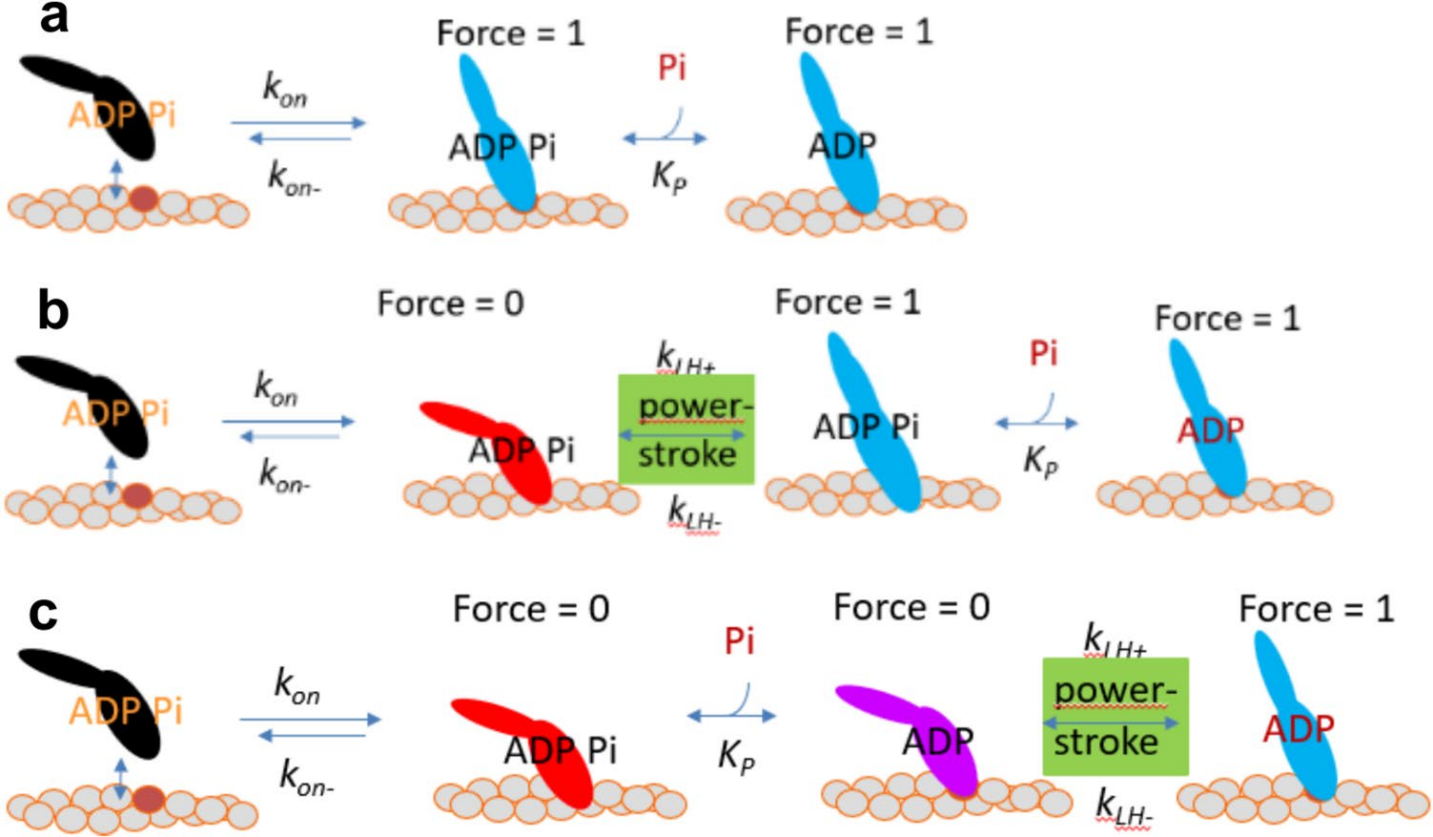
Kinetic schemes to account for effects of varied [Pi] without explicit inclusion of cross-bridge elasticity. **a** Scheme where force is generated coincident with transfer from an unattached or weakly actin-bound state into a strongly actin-bound state. The subsequent Pi-release is assumed to be a rapid equilibrium. Similar to a scheme used previously (Kawai and Halvorson 1991; Dantzig et al. 1992; Tesi et al. 2000; Kawai et al. 2021) to account for different effects of varied [Pi]. **b** Modified version of scheme in a with addition of a transient strongly bound pre-power-stroke cross-bridge state before force generation and Pi-release. **c** Modified scheme with fast Pi-release equilibrium before a fast power-stroke. Cross-bridge detachment by ADP-release and ATP-rebinding neglected in all schemes. The rate constants and equilibrium constants were taken as: k_on_ = 100 s^−1^, k_on-_ = 100 s^−1^, k_LH+_ = 5000 s^−1^, k_LH-_ = 5000 s^−1^, and K_P_ = 10 mM (Pi-release rate constant: 3000 s^−1^). See text for justification of the numerical values

Inspired by previous work (Dantzig et al. 1992; Tesi et al. 2000; Stehle 2024) (see also reviews by (Stehle and Tesi 2017; Kawai 2025), we first attempt to model the Pi-transients and the redevelopment of force from zero tension in terms of simple kinetic schemes (Fig. 1a-c). In these schemes, only strain-independent average rate constants are assumed and different binary force levels (0 or 1) are assigned to each biochemical state. I consider three versions of the schemes. One of these (Fig. 1a) is similar to one that has been previously suggested to account for the [Pi]-dependence of the Pi-transient and other phenomena (Kawai and Halvorson 1991; Dantzig et al. 1992; Tesi et al. 2000; Ranatunga et al. 2002; Ranatunga 2010; Kawai et al. 2021; Stehle 2024). It assumes that force-generation is effectively coincident with strong stereospecific attachment of myosin. This either means transfer of the cross-bridges from detached states or from non-stereospecific weakly bound states (with zero force) in rapid equilibrium with detached states. In the model in Fig. 1a, the force-generating transition is conincident with the rate limiting actin-myosin attachment step and occurs prior to Pi-release. A second model (Fig. 1b) assumes that a rapid force-generating transition, similar to that proposed by (Huxley and Simmons 1971) or (Eisenberg and Hill 1978), occurs immediately after cross-bridge attachment but before Pi-release. A similar scheme was used by Muretta et al. (Muretta et al. 2015) to interpret their transient kinetics results suggesting that a major power stroke component occurs before Pi-release during actomyosin turnover in solution. A scheme similar to that in Fig. 1b was also found to account for ensemble-averaged ultra-fast force spectroscopy data from single cardiac myosin motor domains interacting with actin at varied [Pi] (Woody et al. 2019). Finally, in a third kinetic scheme (Fig. 1c), Pi-release is assumed to occur in attached cross-bridges before the force-generating transtion. Schemes similar to those in Fig. 1 were included among those tested by (Stehle 2024) with focus on coupled effects of varied [Pi] on k_tr_ and isometric force in cardiac myofibrils. Cross-bridge cycling by detachment via re-binding of ATP is neglected (Fig. 1a–c) in our analysis using the kinetic schemes.

In all schemes, the actual Pi-release step from the active site is assumed to be a rapid equilbrium with transition rate constants (> 1000 s^−1^), appreciably faster than k_on_. This has been directly suggested by previous experimental findings (Dantzig et al. 1992) and is also required to account for negligible effects of the Pi-release step on the maximum velocity of shortening (Moretto et al. 2022). There is convincing evidence that the latter contractile parameter is primarily determined by the ADP release rate and subsequent ATP induced actomyosin detachment rate (Siemankowski et al. 1985; Nyitrai et al. 2006) (reviewed by (Rassier and Månsson 2025)). Also the power-stroke is assumed to be very fast in the schemes in Fig. 1b, c. This is consistent with the very fast rate (Linari et al. 2009) of the tension recovery following fast length steps imposed on muscle cells.

Approximately consistent with the mechanokinetic model below (also initially assumed by (Huxley and Simmons 1971)), the equlibrium constant for the power-stroke is set to 1 in Fig. 1b, c. The rate constant for cross-bridge attachment k_on_ as well as its reversal k_on-_ in simulations using kinetic schemes, are approximated from data for fast skeletal muscle at 20°C (Dantzig et al. 1992), setting k_on_ = k_on-_ = 100 s^−1^. These values may overestimate the real rate constants in Pi-transients in response to increased [Pi], because these transients in the experiments might have been speeded up by non-uniform sarcomere behavior as later suggested from experiments on cardiac myofibrils (Stehle 2017). Keeping this possibility in mind, I still use approximate values of k_on_ and k_on-_ suggested in (Dantzig et al. 1992) to illustrate key differences in predictions between the different kinetic schemes in Fig. 1. These numerical values of k_on_ and k_on-_ are higher than the corresponding average values in the mechanokinetic model below, explaining faster Pi-transients and rate of redevelopment of force from zero using the kinetic schemes. Numerical values of all rate constants in the simulations using the kinetic schemes of Fig. 1 are summarized in the legend of Fig. 1.

In order to numerically simulate Pi-transients (associated with k_Pi_) where [Pi] is suddenly changed from 0.5 mM to a higher value, we first simulated the rate of force redevelopment (rise of force from zero, associated with k_tr_) to the steady-state value at 0.5 mM Pi. This allowed us to obtain a set of initial values for all states in the given scheme. The Pi-transients where then simulated by running the numerical computations with these steady-state values for 0.5 mM Pi as initial values at the high [Pi]. The force redevelopment was simulated with all cross-bridges initially assumed to reside in the detached state with ADP and Pi in the active site (left-most state in Fig. 1).

### Mechanokinetic model—general

**Fig. 2:**
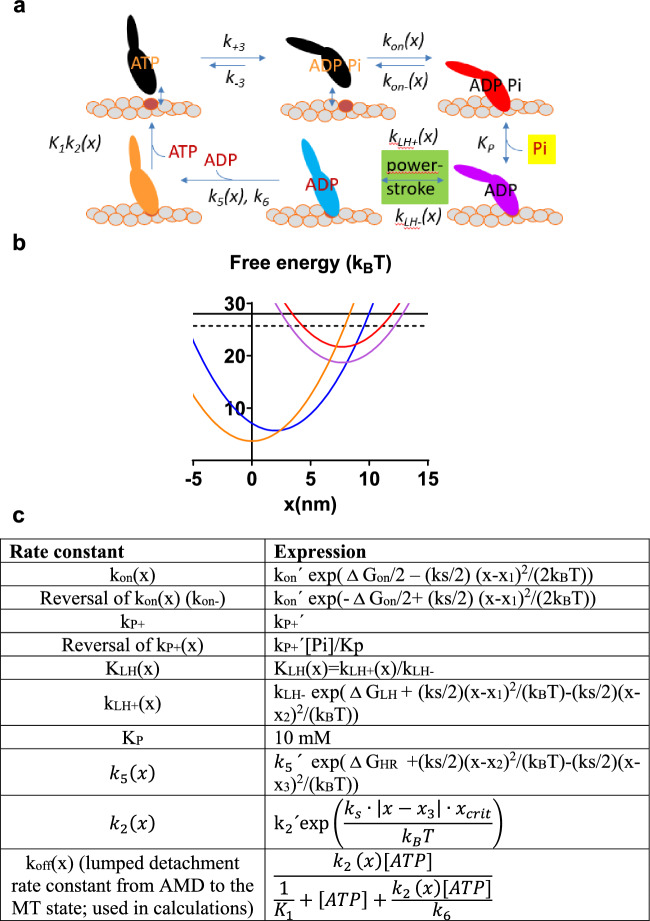
Mechanokinetic model. **a** Myosin (M) cross-bridge states either detached from actin (“A”; black) or attached in different stereospecifically bound states. All states exist at different elastic strains. Myosin either has MgATP (ATP), MgADP (ADP), both MgADP and Pi (P) or no substrate or product in the active site. The subscripts _L_ and _H_ refer to low and high force respectively. I.e. the AMD_L_ to the AMD_H_ transition is the main force-generating transition, the power stroke. The argument (x) indicates strain-dependence of rate constants. **b** Free energy of different states plotted against the position coordinate x. **c** The x-dependence of rate constants and equilibrium constants in the model in **a**, **b**. Note, in some cases the rate constants have one given, constant value for all x. The parameter values in the equations in the right column are given in Tables 1 and 2 unless otherwise specified in the text. MgATP is abbreviated as ATP in **c**

We next modelled the Pi-transients using a full mechanokinetic model (Fig. 2a–c) where effects of cross-bridge elasticity are explicitly taken into account. This means that the free-energy diagrams for each attached biochemical cross-bridge state (Fig. 2a, b) has an elastic contribution (Hill 1974; Eisenberg and Hill 1978; Eisenberg and Greene 1980) and that several rate constants are strain-dependent. The functions defining the strain-dependence are given in Fig. 2c. A position coordinate, x, quantifying the strain, is defined as the distance between the closest actin filament binding site and a myosin head. The coordinate is defined such that x = 0 nm corresponds to the distance where the myosin head binds in the rigor (AM) state at its minimum free energy (Fig. 2). The main force-generating transition in the model in Fig. 2 is the AMD_L_ to AMD_H_ transition that is associated with a swing of the lever-arm during muscle shortening. This is related to the key force-generating swing of the cross-bridge proposed by Huxley and Simmons (Huxley and Simmons 1971) or, more precisely, the force-generating transition suggested by Eisenberg and Hill (Eisenberg and Hill 1978). Below I will denote this transition as the power stroke along with common use in biochemical and biophysical literature (reviewed in Geeves 2016; Robert-Paganin et al. 2020; Rassier and Månsson 2025)). The force in each state is calculated from the probability of that state at each given strain multiplied by the gradient, ks(x − x_i_), of the free energy profile. Here, ks is the cross-bridge stiffness and x_i_ is the x-value where the given state has its minimum free energy. The total observed force from a muscle or myofibril is the total number of cross-bridges per half-sarcomere times the sum of the force attributed to all cross-bridge states averaged over the actin filament periodicity of 36 nm.

In the mechanokinetic model defined in Fig. 2 we use parameter values for fast skeletal muscle at 25–30 °C (Tables 1, 2) that we arrived at previously (Månsson 2016, 2020, 2021; Moretto et al. 2022). We assume a Hookean cross-bridge elasticity and a uniform x-distribution of the myosin heads relative to the myosin binding sites, separated by 36 nm along the actin filaments.

**Table 1:** Parameter values determining shape of free energy diagrams for simulation of contractile properties of fast mammalian skeletal muscle using mechanokinetic model

Parameter	Numerical value
*x-values for positions of free energy minima of different states*	
x_1_ (AMDP_,_ AMD_L_)	7.7 nm
x_2_ (AMD_H_)	2.0 nm
x_3_ (AM, AMD)	0 nm
*Differences in free energies between neighboring states*	
$\Delta$G_on_ (MDP-AMDP)	4 k_B_T
$\Delta$G_P_ (AMDP-AMD_L_)	-k_B_T ln([Pi]/Kp)
$\Delta$G_LH_ (AMD_L_-AMD_H_)	14 k_B_T
$\Delta$G_HR_ (AMD_H_-AMD/AM)	2 k_B_T
$\Delta \mathop G\nolimits_{ATP}$	13.1 + ln ([MgATP]/ ([MgADP][Pi]) k_B_T (28 k_B_T)
*Cross-bridge stiffness*	
ks	2.8 pN/nm

The parameter values are estimated primarily from two-headed myosin motor fragments from fast skeletal muscle of the rabbit at 25–30 °C (see references in text for details)

**Table 2:** Parameter values defining rate functions and kinetic constants for simulation of contractile properties of fast mammalian muscle using mechanokinetic model

Parameter	Numerical value
k_+3_ + k_−3_ (Recovery stroke + hydrolysis)	220 s^−1^
K_3_	10
k_on_′	20 s^−1^
k_P+_′	10,000 s^−1^
k_LH-_	6000 s^−1^
k_5_′	2000 s^−1^
K_P_	10 mM
x_crit_	0.6 nm
k_6_	5000 s^−1^
Physiological [Pi]	0.5 mM
K_1_	1.7 mM^−1^
k_2_′	2000 s^−1^

The parameter values are primarily from two-headed myosin motor fragments from fast skeletal muscle of rabbit at 25–30 °C (see references in text for details)

Steady state isometric cross-bridge distributions with respect to the position variable x of the states in Fig. 2a were approximated by solution of the following set of differential equations in the state probabilities at varied x (Eqs. 1–6) and very low velocity (v ≤ 0.7 nm/s < 0.007% of maximum shortening velocity).

1 $$\frac{{d\left[ {MT} \right]}}{dx} = \left( { - {\text{k}}_{{ + {3}}} \left[ {{\text{MT}}} \right] + {\text{k}}_{{ - {3}}} \left[ {{\text{MDP}}} \right] + {\text{k}}_{{{\text{off}}}} \left[ {{\text{AMD}}} \right]} \right)/{\text{v}}$$

2 $$\frac{{d\left[ {MDP} \right]}}{dx} = \left( {{\text{k}}_{{ + {3}}} \left[ {{\text{MT}}} \right] + {\text{k}}_{{{\text{on}} - }} \left( {\text{x}} \right)\left[ {{\text{AMDP}}} \right] - \left( {{\text{k}}_{{{\text{on}}}} \left( {\text{x}} \right) + {\text{k}}_{{ - {3}}} } \right)\left[ {{\text{MDP}}} \right]} \right)/{\text{v}}$$

3 $$\frac{{d\left[ {AMDP} \right]}}{dx} = \left( {{\text{k}}_{{{\text{on}}}} \left( {\text{x}} \right)\left[ {{\text{MDP}}} \right] + \left( {{\text{k}}_{{{\text{P}} + }} \left[ {{\text{Pi}}} \right]/{\text{K}}_{{\text{P}}} } \right)\left[ {{\text{AMD}}_{{\text{L}}} } \right] - \left( {{\text{k}}_{{{\text{on}} - }} \left( {\text{x}} \right) + {\text{k}}_{{{\text{P}} + }} } \right) \, \left[ {{\text{AMDP}}} \right]} \right)/{\text{v}}$$

4 $$\frac{{d\left[ {AMD_{L} } \right]}}{dx} = ({\text{k}}_{{{\text{LH}} - }}\left[ {{\text{AMD}_\text{H}}} \right] + {\text{ k}}_{{{\text{P}} + }} \left[ {{\text{AMDP}}} \right] - \left(\!\!{{\text{ k}}_{{{\text{LH}} + }} \left( {\text{x}} \right) + {\text{ k}}_{{{\text{P}} + }} \left[ {{\text{Pi}}} \right]/{\text{K}}_{{\text{P}}} } \right)[\text{AMD}_\text{L}])/{\text{v}}$$

5 $$\frac{{d\left[ {AMD_{H} } \right]}}{dx} = ({\text{k}}_{{{\text{LH}} + }} \left( {\text{x}} \right)[\text{AMD}_\text{L}]- ( {{\text{k}}_{{{\text{LH}} - }} + {\text{k}}_{{5}} \left( {\text{x}} \right))}[\text{AMD}_\text{L}])/{\text{v}}$$

6 $$\frac{{d\left[ {AMD} \right]}}{dx} = ({\text{k}}_{{5}} \left( {\text{x}}\right)[\text{AMD}_\text{L}]  { - {\text{k}}_{{{\text{off}}}} \left( {\text{x}} \right)}[{\text{AMD}}])/{\text{v}}$$

The probability (indicated by []) of a given state in the differential Eqs. 1–6 is a function of x but the argument (x) is omitted for clarity. The differential equations for obtaining steady-state solutions at a given velocity were numerically solved using a Runge–Kutta Fehlberg algorithm in the program Simnon (for details, see (Månsson 2019; Moretto et al. 2022; Månsson and Rassier 2022)). The numerical computations started at 14–15 nm and ran in the negative x-direction to cover all x-values with the state probabilities differing from their initial values at high x. The latter initial values were [AMDP] = [AMD_L_] = [AMD_H_] = [AM] = 0 whereas [MT]  and [MDP] were set to 0.1 and 0.9, respectively approximating K_3_ = 10 (Table 2). To avoid instabilities in the computations, values of rate functions larger than r_max_ = 300 000 s^−1^ or smaller than r_min_ = 0.001 s^−1^ were set to either of these values or 0 (for r_min_).

### Simulations of Pi-transients and force redevelopment using mechanokinetic model

#### Pi-transients

Pi-transients were simulated starting at 0.5 mM Pi, assuming instantaneous increases of [Pi] to either 5, 10, 25 or 50 mM (final concentrations). A slow component of the Pi-transients is due to cross-bridges in the AMD_L_ and AMDP states at high x whereas a fast component is due to cross-bridges in the AMD_H_ state. We started by calculating the difference ΔF(x) in steady-state isometric force between 0.5 mM Pi and the final Pi concentration at equidistant x-values (x = 5, 5.25…14, 14.25 nm). We could then isolate one slow, ΔF_slow_(x), and one fast, ΔF_fast_(x)) component. Explicitly,

7 $$\Delta {\text{F}}_{{{\text{slow}}}} \left( {\text{x}} \right) = {\text{ks }}\left( {{\text{x}} - {\text{x}}_{{1}} } \right) \, \left( {\left( {\left[ {{\text{AMDP}}} \right]\left( {\text{x}} \right) + \left[ {{\text{AMD}}_{{\text{L}}} } \right]}\left( {\text{x}} \right) \right)_{{0.{5}}} - \left( {\left[ {{\text{AMDP}}} \right]\left( {\text{x}} \right) + \left[ {{\text{AMD}}_{{\text{L}}} } \right]} \left( {\text{x}} \right) \right)_{{\left[ {{\text{Pi}}} \right] - {\text{end}}}} } \right)$$

and

8 $$\Delta {\text{F}}_{{{\text{fast}}}} \left( {\text{x}} \right) = {\text{ks }}\left( {{\text{x}} - {\text{x}}_{{2}} } \right) \, \left( {\left[ {{\text{AMD}}_{{\text{H}}} } \right] \left( {\text{x}} \right) _{{0.{5}}} - \, \left[ {{\text{AMD}}_{{\text{H}}} } \right] \left( {\text{x}} \right)_{{\left[ {{\text{Pi}}} \right] - {\text{end}}}} } \right)$$

where the indices “0.5” and “[Pi]-end” stand for [Pi] = 0.5 mM and the final Pi-concentration, respectively.

We then simulated a Pi-transient (F_Pi_(t)) as:

9 $${\text{F}}_{{{\text{Pi}}}} \left( {\text{t}} \right) = {\text{F}}_{{0.{5}}} {-}(\Delta {\text{FP}}_{{{\text{slow}}}} + \Delta {\text{FP}}_{{{\text{fast}}}} ) + \Delta {\text{FP}}_{{{\text{slow}}}} {{\left( {\mathop \sum \limits_{{{\text{i}} = 0}}^{20} {\Delta F}_{{{\text{slow}}}} \left( {\text{x}} \right){\text{e}}^{{ - {\text{k}}_{{{\text{Pi}}}}^{{{\text{slow}}}} \left( {\text{x}} \right){\text{ t}}}} } \right)} \mathord{\left/ {\vphantom {{\left( {\mathop \sum \limits_{{{\text{i}} = 0}}^{20} {\Delta F}_{{{\text{slow}}}} \left( {\text{x}} \right){\text{e}}^{{ - {\text{k}}_{{{\text{Pi}}}}^{{{\text{slow}}}} \left( {\text{x}} \right){\text{ t}}}} } \right)} {\left( {\mathop \sum \limits_{{{\text{i}} = 0}}^{20} {\Delta F}_{{{\text{slow}}}} \left( {\text{x}} \right)} \right)}}} \right. \kern-0pt} {\left( {\mathop \sum \limits_{{{\text{i}} = 0}}^{20} {\Delta F}_{{{\text{slow}}}} \left( {\text{x}} \right)} \right)}} + \Delta {\text{FP}}_{{{\text{fast}}}} {{\left( {\mathop \sum \limits_{{{\text{i}} = 23}}^{42} {\Delta F}_{{{\text{fast}}}} \left( {\text{x}} \right){\text{e}}^{{ - {\text{k}}_{{{\text{Pi}}}}^{{{\text{fast}}}} \left( {\text{x}} \right){\text{ t}}}} } \right)} \mathord{\left/ {\vphantom {{\left( {\mathop \sum \limits_{{{\text{i}} = 23}}^{42} {\Delta F}_{{{\text{fast}}}} \left( {\text{x}} \right){\text{e}}^{{ - {\text{k}}_{{{\text{Pi}}}}^{{{\text{fast}}}} \left( {\text{x}} \right){\text{ t}}}} } \right)} {\left( {\mathop \sum \limits_{{{\text{i}} = 23}}^{42} {\Delta F}_{{{\text{fast}}}} \left( {\text{x}} \right)} \right)}}} \right. \kern-0pt} {\left( {\mathop \sum \limits_{{{\text{i}} = 23}}^{42} {\Delta F}_{{{\text{fast}}}} \left( {\text{x}} \right)} \right)}}$$

**Fig. 3:**
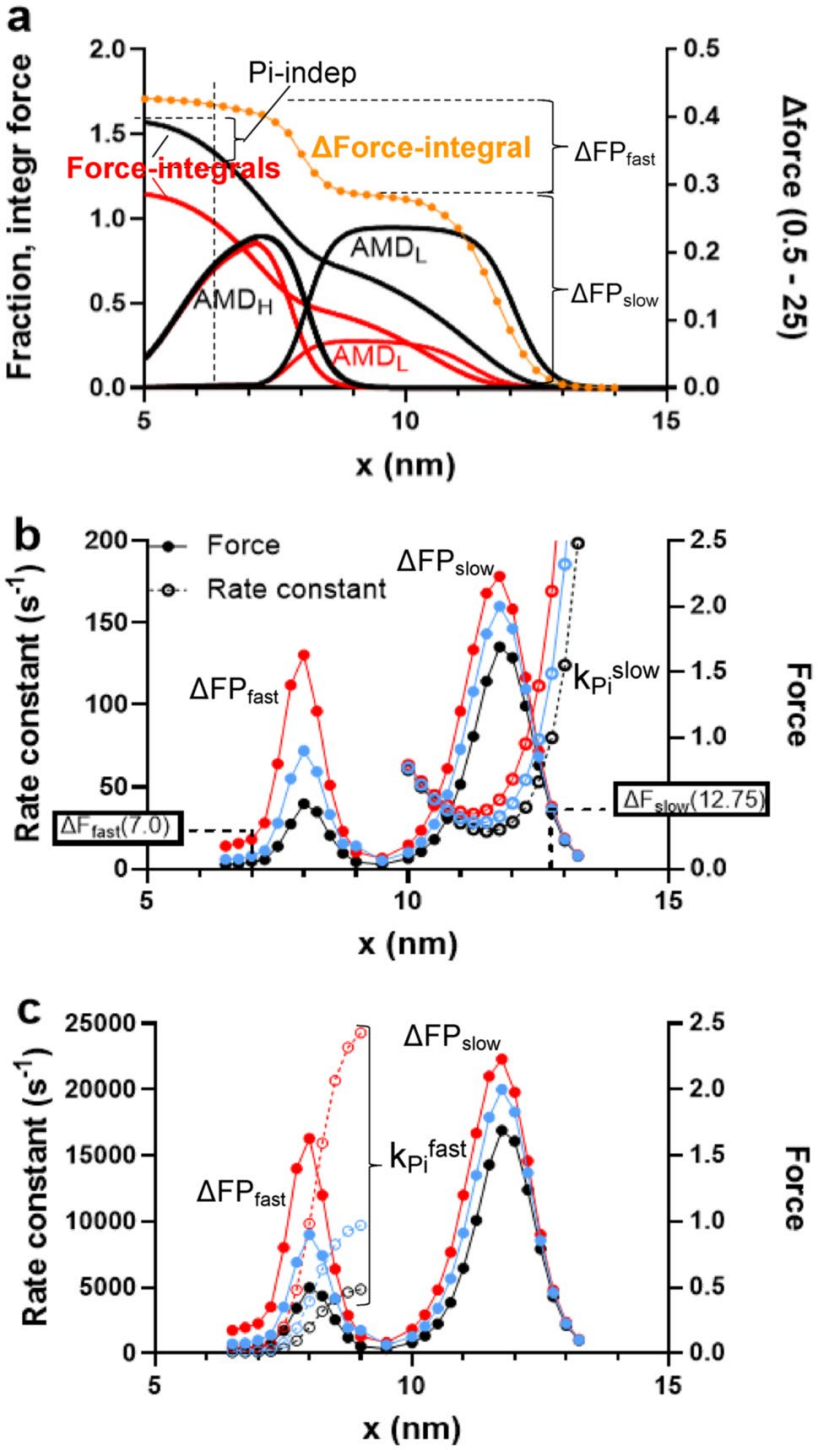
Basis for complex Pi-transients in mechanokinetic model. **a** Cross-bridge distributions (“AMD_L_” and “AMD_H_”; probability [left vertical axis] of each state vs x) and integral of isometric force (“force-integrals”; left vertical axis; pN/head) associated with these distributions at 0.5 mM (black) and 25 mM Pi (red). Also shown (orange; right vertical axis; pN/head) is the difference between these integrals clearly revealing two components (one slow “ΔFP_slow_” at x > 9 nm and one fast “ΔFP_fast_” in the range x≈ [6, 9] nm) making up the force decrease upon increased [Pi]. Integration of force starts at x = 14 nm and runs in the negative x-direction. **b** Two components of force-decrease (right vertical axis) upon increased [Pi] depicted differently than in **a**, by plot of difference in steady-state force (pN/(36 nm)) vs x between 0.5 mM and higher [Pi] (black: 5 mM; blue: 10 mM; red 25 mM). Force-difference data superimposed on rate constant (left vertical axis) for Pi-induced reversal of attachment from the AMD_L_ to the MDP state (ΔFP_slow_). Examples are given to indicate how we obtain the values $\Delta {\text{F}}_{\text{fast}}\left(\text{x}\right)$ and $\Delta {\text{F}}_{\text{slow}}\left(\text{x}\right)$ in Eq. 9 as well as the rate constant k_Pi_^slow^(x). **c** The two components of [Pi]-dependent force-decrease (right vertical axis) from b replotted and superimposed on rate constant (left vertical axis) for Pi-induced transitions from the AMD_H_ to the MDP state (ΔFP_fast_). The rate constants k_Pi_.^fast^(x) indicated. Parameter values are given in Tables 1, 2

Here, ΔFP_slow_ and ΔFP_fast_ is the total amplitude of the slow and fast component, respectively of the difference in steady-state force between 0.5 mM Pi and higher [Pi] (cf. Fig. 3a). Further, F_0.5_ is the steady-state isometric force at [Pi] = 0.5 mM with F_Pi_(t) for t—> ∞ being equal to F_0.5_—(ΔFP_slow_ + ΔFP_fast_), i.e. the steady-state force at the higher [Pi] at the end of the transient. The index i is related to the position coordinate x such that x = 14.5–0.25i nm, i.e. i = 0, 20, 23 and 42 correspond to x = 14.5 nm, 9.5 nm, 8.75 nm and 4 nm, respectively. The numerical values for ΔF_slow_(x) and ΔF_fast_(x) at all x-values were obained by numerical solutions (using Simnon) of the steady-state isometric force levels vs x (Eqs. 7, 8) at both 0.5 mM Pi and the final Pi-concentration. The effective x-dependent rate constants at the final Pi-concentration, k_Pi_^slow^(x) and k_Pi_^fast^(x) to be inserted in Eq. 9, are given in Fig. 2c and by the following equations:

10 $${\text{k}}_{{{\text{Pi}}}}^{{{\text{slow}}}} \left( {\text{x}} \right) = {\text{k}}_{{{\text{on}}}} {\_} \left( {\text{x}} \right)\left[ {{\text{Pi}}} \right]/\left( {{\text{Kp}}\left( {{1} + {\text{K}}_{{{\text{LH}}}} \left( {\text{x}} \right)_{{}} } \right) + \left[ {{\text{Pi}}} \right]} \right) + {\text{k}}_{{{\text{on}}}} \left( {\text{x}} \right)$$

11 $${\text{k}}_{{{\text{Pi}}}}^{{{\text{fast}}}} \left( {\text{x}} \right) = {\text{k}}_{{{\text{p}} + }} \left( {\text{x}} \right)\left( {\left[ {{\text{Pi}}} \right]/{\text{Kp}}} \right)\left( {{1}/{\text{ K}}_{{{\text{LH}}}} \left( {\text{x}} \right)_{{}} } \right)/\left( {{1}/{\text{ K}}_{{{\text{LH}}}} \left( {\text{x}} \right) \, + {1}} \right)$$

The meanings of the symbols used in Eqs. 9–11 are indicated in Fig. 3.

#### Redevelopment of isometric force

As justified below (Results), the rate of redevelopment of isometric force F_tr_(t) only has slow components because the power stroke (the AMD_L_ to AMD_H_ transition) occurs after the rate limiting step. Additionally, as also justified below, the rate of force redevelopment has one Pi-dependent and one Pi-independent component.

12 $${\text{F}}_{{{\text{tr}}}} \left( {\text{t}} \right) = {\text{Fint}}_{P} {{\left( {\mathop \sum \limits_{{{\text{i}} = 0}}^{32} F_{P}^{SS} \left( {\text{x}} \right)\left( {1 - {\text{e}}^{{ - {\text{k}}_{{{\text{Pi}}}}^{slow} \left( {\text{x}} \right){\text{t}}}} } \right)} \right)} \mathord{\left/ {\vphantom {{\left( {\mathop \sum \limits_{{{\text{i}} = 0}}^{32} F_{P}^{SS} \left( {\text{x}} \right)\left( {1 - {\text{e}}^{{ - {\text{k}}_{{{\text{Pi}}}}^{slow} \left( {\text{x}} \right){\text{t}}}} } \right)} \right)} {\left( {\mathop \sum \limits_{{{\text{i}} = 0}}^{32} F_{P}^{SS} \left( {\text{x}} \right)} \right)}}} \right. \kern-0pt} {\left( {\mathop \sum \limits_{{{\text{i}} = 0}}^{32} F_{P}^{SS} \left( {\text{x}} \right)} \right)}} + {\text{Fint}}_{Pind} {{\left( {\mathop \sum \limits_{{{\text{i}} = 33}}^{42} F_{Pind}^{SS} \left( {\text{x}} \right)\left( {1 - {\text{ e}}^{{ - {\text{k}}_{{{\text{on}}}}^{{}} \left( {\text{x}} \right){\text{t}}}} } \right)} \right)} \mathord{\left/ {\vphantom {{\left( {\mathop \sum \limits_{{{\text{i}} = 33}}^{42} F_{Pind}^{SS} \left( {\text{x}} \right)\left( {1 - {\text{ e}}^{{ - {\text{k}}_{{{\text{on}}}}^{{}} \left( {\text{x}} \right){\text{t}}}} } \right)} \right)} {\left( {\mathop \sum \limits_{{{\text{i}} = 33}}^{42} F_{Pind}^{SS} \left( {\text{x}} \right)} \right){ }}}} \right. \kern-0pt} {\left( {\mathop \sum \limits_{{{\text{i}} = 33}}^{42} F_{Pind}^{SS} \left( {\text{x}} \right)} \right){ }}}$$

here, ${\text{Fint}}_{P}$ and ${\text{Fint}}_{Pind}$ are the [Pi]-dependent and [Pi]-independent components of steady-state isometric force at the given [Pi]. As justified under Results, we estimate the values from the integral of the steady-state cross-bridge force in the range [6.5, 14.5] nm (${\text{Fint}}_{P})$ and [4, 6.25] nm (${\text{Fint}}_{Pind})$, respectively. The quantity ${\text{F}}_{\text{P}}^{\text{SS}}\left(\text{x}\right)\text{ is given by }{\text{F}}^{\text{SS}}\left(\text{x}\right)\text{ for x}\ge 6.5\text{ nm and }{\text{F}}_{\text{Pind}}^{\text{SS}}\left(\text{x}\right)={\text{F}}^{\text{SS}}\left(\text{x}\right)\text{ for x}<6.5\text{ nm}$. These are the steady-state isometric force levels at the given x-values. The quantity ${\text{k}}_{\text{Pi}}^{slow}\left(\text{x}\right)$ is given by Eq. 10 whereas ${\text{k}}_{\text{on}}\left(\text{x}\right)$ is given in Fig. 2c. See also Fig. 3. The position coordinate x relates to the summation index, i as: x = 14.5–0.25i nm, i.e. i = 0, 32, 33 and 42 correspond to x = 14.5 nm, 6.5 nm, 6.25 nm and 4 nm, respectively. The simulation of the redevelopment of force is carried out under the simplifying approximation of the following initial values:

13 $$\left[ {{\text{AMDP}}} \right]\left( {\text{x}} \right) = \left[ {{\text{AMD}}_{{\text{L}}} } \right]\left( {\text{x}} \right) = \left[ {{\text{AMD}}_{{\text{H}}} } \right]\left( {\text{x}} \right) = \left[ {{\text{AM}}} \right]\left( {\text{x}} \right) = \left[ {{\text{MT}}} \right]\left( {\text{x}} \right) = 0{\text{ whereas }}\left[ {{\text{MDP}}} \right]\left( {\text{x}} \right) = {\text{1 for all x}}$$

### Simplified analysis of effects of multistep Pi-release and rebinding

**Fig. 4:**
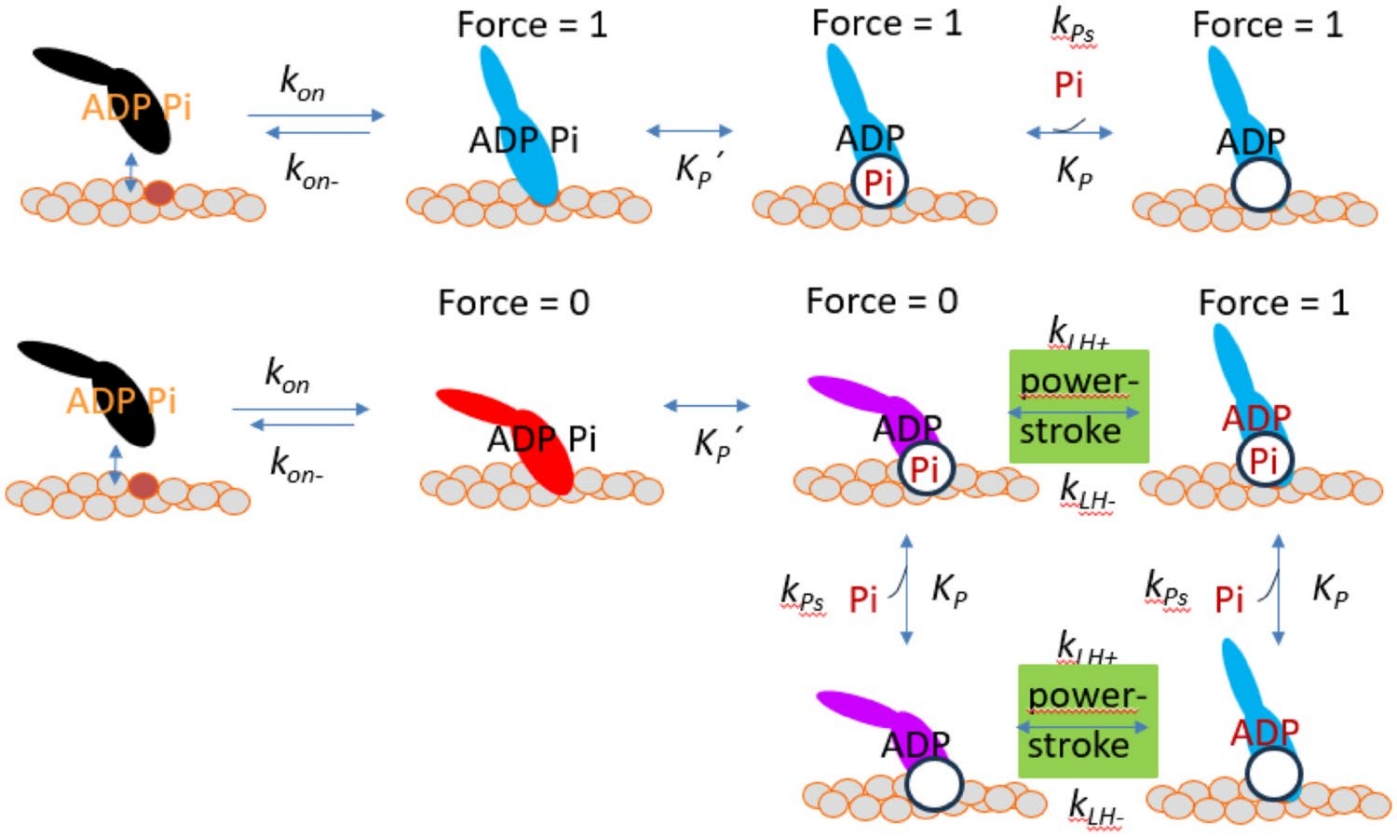
Model for simulation of the effects of multistep Pi-release on Pi-transients and the force redevelopment during contraction at varied [Pi]. The schemes in Fig. 1a and c have been expanded with additional states to integrate a secondary Pi-binding site on myosin outside the active site (circle). Pi is first assumed to be rapidly transferred (rate constant 3000 s^−1^) from the active site to the secondary site governed by equlibrium constant Kp′ = 1. Pi is then assumed to be released from the secondary site with rate constant k_Ps_ of 100 or 200 s^−1^ varied as described below with and K_P_ = 10 mM. The other rate constants are as follows (from Fig. 1): k_on_ = 100 s^−1^, k_on-_ = 100 s^−1^, k_LH+_ = 5000 s^−1^, k_LH-_ = 5000 s^−1^. Both top and bottom schemes are assumed to operate in parallel to a degree proportional to the amplitude of the fast and slow components of force redevelopment and Pi-transients in the mechanokinetic scheme as described in greater detail in the text

We first approximated the mechanokinetic model with two parallel kinetic schemes with the aim to capture the Pi-dependent fast and slow components of the Pi-transients (cf. Eq. 9). The numerical computations (using Simnon), to obtain Pi-transients with initial [Pi] = 0.5 mM followed by instantaneous exchange to higher [Pi], used initial values for the state probabilities obtained by steady-state solution for isometric contraction of each of the schemes in Fig. 4 at [Pi] = 0.5 mM. The numerical solutions were then run at the high [Pi] with these initial values. The slow component was simulated by the kinetic scheme in the top of Fig. 4 (similar to scheme in Fig. 1a supplemented with a secondary Pi-binding site with relatively slow Pi-release and rebinding). The fast component of the Pi-transient, on the other hand, was simulated by the bottom scheme in Fig. 4 (similar to that in Fig. 1c but, again supplemented with a secondary Pi-binding site with slow Pi-release and re-binding). The amplitudes of the two components operating in parallel were taken as the amplitudes of the slow ($\Delta {FP}_{slow}$) and fast ($\Delta {FP}_{fast}$) components, respectively, directly calculated from the mechanokinetic model at the given [Pi] (see Results below). The rate constants, on the other hand, that were inserted in the computations were those used in the kinetic schemes in Fig. 1 supplemented with a slow Pi-dissociation rate constant k_Ps_ from the secondary site (details in legend of Fig. 4). The force redevelopment was also simulated using the schemes in Fig. 4 but supplemented with one additional Pi-independent component. First, the calculations were performed for each of the two schemes in Fig. 4 with initial values of [MDP] for the given scheme set to 1 whereas all other state probabilities were set to 0. After these calculations, the amplitudes of the two Pi-dependent components were assigned to the component of force contribution at x > 9 nm (top scheme in Fig. 4) and 6.5 nm ≤ x ≤ 9 nm (bottom scheme in Fig. 4) for the given [Pi] obtained using the mechanokinetic model. Additionally a [Pi]-independent component was added. The latter component was a single exponential with rate constant k_on_ = 100 s^−1^ and amplitude given by the force contribution for x < 6.5 nm calculated using the mechanokinetic model.

## Results

### Simulations of Pi-transients using kinetic schemes

The equations for change in the fraction of strongly attached cross-bridges with time ($As\left(t\right))$ for the kinetic schemes in Fig. 1a–c are given below (Eqs. 14, 15) With all cross-bridges initially in the MDP state (assumed at the onset of contraction from zero force and force redevelopment) the following equation applies:

14 $$As\left(t\right)=\frac{{k}_{on}}{{k}_{AS}}\left(1-{e}^{-{k}_{AS}t}\right)$$

here, k_on_ is the attachment rate constant and k_AS_ is a function of k_on_ and other parameter values (see Table 3). Because force, in all the schemes in Fig. 1a–c, is proportional to $As\left(t\right)$ and all other rate constants are assumed to be much greater than (k_on_ + k_on-_) the rate constant k_AS_ would approximate the rate constant, k_tr_ for the isometric tension rise at full activation such as in force redevelopment.

**Table 3:** Kinetic schemes—analytic solutions

Scheme	Rate constant k_AS_^a^	Fraction fast phase (ΔF_fast_) of force drop during Pi-transient	k_tr_ vs k_Pi_
Figure 1a	$\frac{{k}_{on-}[Pi]}{\left[Pi\right]+{K}_{P}}+kon$	Only one phase (100%)	k_tr_ = k_Pi_
Figure 1b	$\frac{{k}_{on-}[Pi]}{\left[Pi\right]+{{K}_{LH}(\left[Pi\right]+K}_{P})}+kon$	${{A}_{SS}^{start}( {C}_{start}K}_{LH}\left(1+\frac{{K}_{P}}{{\left[Pi\right]}_{start}}\right)-{ {C}_{end}K}_{LH}\left(1+\frac{{K}_{P}}{{\left[Pi\right]}_{end}}\right))/{{(A}_{SS}^{start}( {C}_{start}K}_{LH}\left(1+\frac{{K}_{P}}{{\left[Pi\right]}_{start}}\right))-{A}_{SS}^{end}({ {C}_{end}K}_{LH}\left(1+\frac{{K}_{P}}{{\left[Pi\right]}_{end}}\right) )$	k_tr_ < < k_Pi_
Figure 1c	$\frac{{k}_{on-}[Pi]}{\left[Pi\right]+{{K}_{P}(1+K}_{LH})}+kon$	$(\frac{{A}_{SS}^{start}{K}_{LH}{K}_{P}}{{\left[Pi\right]}_{start}+{{K}_{P}(1+K}_{LH})}-\frac{{A}_{SS}^{start}{K}_{LH}{K}_{P}}{{\left[Pi\right]}_{end}+{{K}_{P}(1+K}_{LH})})/$ $(\frac{{A}_{SS}^{start}{K}_{LH}{K}_{P}}{{\left[Pi\right]}_{start}+{{K}_{P}(1+K}_{LH})}-\frac{{A}_{SS}^{end}{K}_{LH}{K}_{P}}{{\left[Pi\right]}_{end}+{{K}_{P}(1+K}_{LH})})$	k_tr_ < < k_Pi_

^a^The steady state concentration, As^ss^ vs [Pi] is given by the ratio k_on_/k_AS_

^b^${C}_{start}=\frac{{[Pi]}_{start}}{{\left[Pi\right]}_{start}+{{K}_{LH}({\left[Pi\right]}_{start}+K}_{P})}$; ${C}_{end}=\frac{{[Pi]}_{end}}{{\left[Pi\right]}_{end}+{{K}_{LH}({\left[Pi\right]}_{end}+K}_{P})}$ where the subscript “start” and “end” refer to [Pi] before and after sudden change to initiate a Pi-transient. ${A}_{SS}^{start}$ and ${A}_{SS}^{end}$ refer to the steady-state proportion of strongly bound cross-bridges for the [Pi] before and after the sudden change, respectively

The alteration in the fraction of strongly attached cross-bridges after a sudden change in [Pi] is also governed by the rate constant k_AS_ in all schemes in Fig. 1a–c according to the following equation:

15 $$As\left(t\right)=\left(\frac{{k}_{on}}{{k}_{AS}^{S}}-\frac{{k}_{on}}{{k}_{AS}^{E}}\right){e}^{-{k}_{AS}t}+\frac{{k}_{on}}{{k}_{AS}^{E}}$$

here, the superscripts S and E denote the value of ${k}_{AS}^{S}$ and ${k}_{AS}^{E}$ before (Start) and after (End) the change in [Pi] in a Pi-transient experiment. The steady state probability of strongly attached states at initial and final [Pi] are given by As_ss_^start^= $\frac{{k}_{on}}{{k}_{AS}^{s}}$ and As_ss_^end^ = $\frac{{k}_{on}}{{k}_{AS}^{E}}$, respectively. The rate constants ${k}_{AS}^{S}$ and ${k}_{AS}^{E}$ are functions of [Pi] (Table 3).

**Fig. 5:**
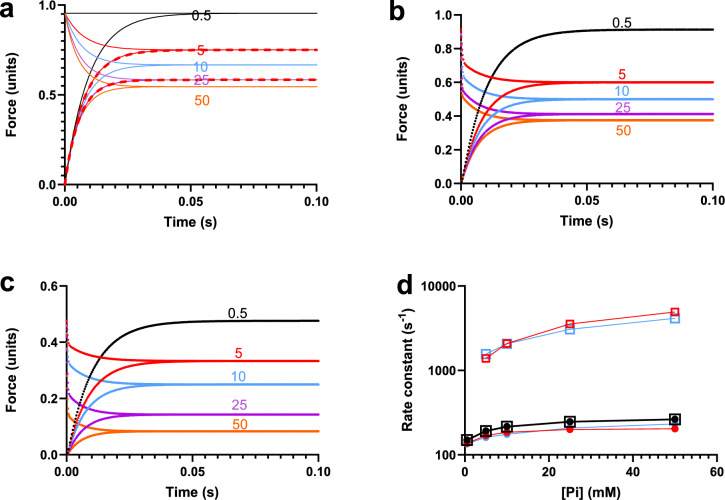
Simulations based on kinetic schemes of isometric tension redevelopment and Pi-transients. **a** Analytical solutions (full lines; Eqs. 14, 15 and Table 3) for the scheme in Fig. 1a with superimposed numerical simulations (dashed lines) based on calculations in Simnon for 5 and 25 mM Pi. **b** Numerical solutions for the scheme in Fig. 1b clearly depicting differences between Pi-transients (double exponential) and force redevelopment from zero (single exponential). Also note the dominance of the very fast phase, particularly at high [Pi]. **c** Numerical solutions for the scheme in Fig. 1c. Also in this case, note differences between Pi-transients and force redevelopment as in **b**. Further, note even greater dominance of the very fast phase than in **b**. **d** Apparent rate constant (1/t_1/2_) for Pi-transient (open squares) and force redevelopment (filled cricles; k_tr_) vs [Pi] for the schemes in Fig. 1a (black), Fig. 1b (red) and Fig. 1c (blue). Note that k_tr_ = k_Pi_ only for the scheme in Fig. 1a. Rate constants in schemes: k_on_ = k_on-_ 100 s^−1^, K_LH_ = 1 (k_LH+_  = k_LH-_ 5000 s^−1^), and k_P+_ = 3000 s^−1^, K_P_ = 10 mM

Equations 14, 15 and the first column in Table 3, describe As(t) for all schemes in Fig. 1 with As(t) in Eq. 14 being proportional to level of force redevelopment from zero. Equation 15, on the other hand, describes the time course for the tension changes (represented by rate constant k_Pi_) in response to a sudden change in [Pi] (Pi-transients) only for the scheme in Fig. 1a, i.e. k_AS_ = k_tr_ = k_Pi_ in this case (Fig. 5a). For the other schemes (Fig. 1b, c), the theoretical Pi-transient is a double exponential function dominated by a fast phase (Table 3 and Fig. 5b, c). The rate constant of the fast phase is determined by the transitions between different strongly attached states. Because these are assumed to be fast equilibria (K_P_ and K_LH_), the rate constant is > tenfold higher than k_AS_. The force redevelopment as well as Pi-transient responses of the different kinetic schemes in Fig. 1 are given in Fig. 5 at varying Pi-concentrations. Composite apparent rate constants, given by 1/half-time (1/t_1/2_) of the tension change as function of [Pi] are plotted in Fig. 5d for both the rate of force redevelopment and the rate of the Pi-transients for each of the kinetic schemes in Fig. 1. Only the scheme in Fig. 1a predicts similar behavior of k_tr_ and k_Pi_. The differences between k_tr_ and k_Pi_ for the other schemes is attributed to the double exponential behavior of the Pi-transient.

The rate constant k_tr_ predicted by all schemes in Fig. 1 increases with [Pi] according to a rectangular hyperbola from 130 to 150 s^−1^ at trace [Pi] to 200–260 s^−1^ at saturating [Pi] (Fig. 5d). Half the maximum Pi-induced increase in rate constant occurs at [Pi] = 10 mM with Kp set to 10 mM. As mentioned above, the [Pi] dependence of k_Pi_ predicted by the scheme in Fig. 1a is the same as the [Pi]-dependence of k_tr_. Also for the schemes in Fig. 1b, c, the apparent k_Pi_ increases with [Pi] but it is more than 10 times higher than k_tr_ (Fig. 5d) due to the large fractional contribution of the fast phase.

### Simulations using mechanokinetic model

Next we moved on from the simple kinetic schemes to instead simulate Pi-transients and the force redevelopment using the mechanokinetic model. We started our analysis by obtaining steady-state solutions of the distributions of the cross-bridge populations and force as a function of x (Fig. 3). In accordance with previous modelling results (Månsson 2021) this simulation suggests that increased [Pi] particularly reduces the occupancy probability of the pre-power-stroke states (e.g. AMD_L_) and to only minor degree that of the post-power-stroke AMD_H_ state. The steady-state population of the pre-power-stroke AMDP state as well as the detached MDP states both increase (not shown) by the increase in [Pi] during isometric contraction. The reason for only a minor reduction in the AMD_H_ state population upon increased [Pi] is that the strong binding-energy (low free energy) in the AMD_H_ state for low values of x limits population of the AMD_L_ state in which Pi-binding is possible.

By observing the force integrals along x (Fig. 3a) at both 0.5 and 25 mM Pi (with integration starting at x = 14 nm and progressing in the negative x-direction) along with their difference at given x-values (orange symbols in Fig. 3a) it is clear that the decrease in steady-state isometric force between 0.5 to 25 mM Pi has two main components that contribute to the Pi-transient. The major component (ΔFP_slow_) is due to loss of cross-bridges from the AMD_L_ and AMDP states to the MDP state upon Pi-binding and reversal of the cross-bridge attachment step. The second, smaller component of force decrease (ΔFP_fast_) is due to reversal of the Pi-release. By mass action it leads to force-reduction due to depletion of the AMD_H_ state for x close to x ≈ x_1_ where the free energy of the AMD_H_ and AMD_L_ states are similar. Notably, only for this minor component is reversal of the power stroke (AMD_H_—> AMD_L_ state) involved.

The two components are apparent (Fig. 3a) from the inflection point in the difference plot (orange dots and lines) between the two integrals of force over x for 0.5 (black) and 25 mM Pi (red) and from the double peaks of the difference in cross-bridge force vs x between 0.5 and 25 mM Pi (Fig. 3b, c). The latter differences are superimposed on the rate constant for the slow (Fig. 3b) and fast (Fig. 3c) component, respectively. The results in Fig. 3 with two distinct components accounting for the difference in steady-state tension between low and high [Pi] suggest a complex response of a mechanokinetic model to changes in [Pi]. During the Pi-transient, one thus expects one slow dominating single exponential component of the force change. This component, ΔFP_slow_, behaves similarly to the kinetic scheme in Fig. 1a but with a distribution of rate constants and force levels. It is due to the Pi-induced detachment from the AMD_L_ state via the AMDP state to the MDP state at high x-values. A smaller fast component, ΔFP_fast_, in the tension response is dominated by a first rapid shift of the equilibrium out of the AMD_H_ to the AMD_L_, and particularly, the AMDP state. This may be followed by slow subsequent detachment into the MDP state. The ΔFP_fast_ component is analogous of the kinetic scheme in Fig. 1c. However, with the rate functions used in the mechanokinetic model, the amplitude of the slow phase of the ΔFP_fast_ component is generally negligible. Thus, to summarize, the tension response of the present mechanokinetic model to a sudden change in [Pi] can be approximated by the sum of two components. The dominant component ΔFP_slow_ is fitted by a single exponential function analogous to the kinetic scheme in Fig. 1a (detachment from the AMD_L_ state at high strain). The other smaller fast component ΔFP_fast_ is analogous to the kinetic scheme in Fig. 1c but with a negligible slow phase. Finally, for the force redevelopment from zero force (rate constant k_tr_) the mechanokinetic model suggests the existence of one additional Pi-independent component (Fig. 3a). This is attributed to the virtually irreversible transition from the MDP over the AMDP and AMD_L_ states to the AMD_D_ state at x < < x_1_ where K_LH_ > > 1. For these x-values, the cross-bridge behaviour according to the mechanokinetic model is similar to that of the kinetic scheme in Fig. 1c if cross-bridge detachment by ADP-release and ATP binding is neglected.

**Fig. 6:**
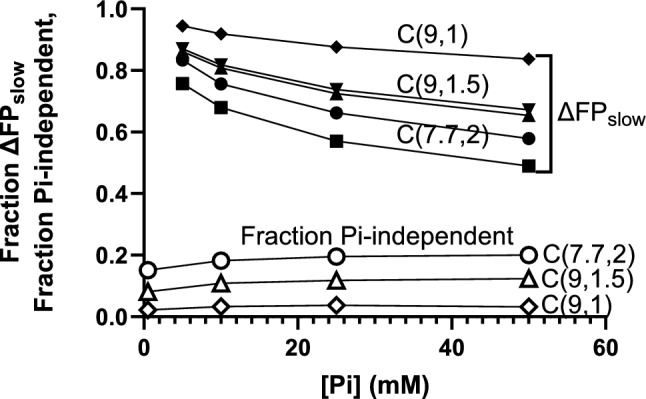
The fraction of slow (ΔFP_slow_) [Pi]-dependent components of Pi-transients (filled symbols) and the fraction of Pi-independent component of the force redevelopment (open symbols) in mechanokinetic model. All parameter values are as in Tables 1, 2 with exceptions as follows: Circles C(7.7, 2), no exception. Squares, ΔG_on_ = 2 k_B_T and k_on_′ = 89 s^−1^. Triangles, x_1_ = 9 nm and x_2_ = 1.5 nm (peak down k_on_′ = 20 s^−1^ peak up k_on_′ = 40 s^−1^; C9,1.5)). Diamonds, x_1_ = 9 nm and x_2_ = 1 nm (C(9,1))

We next evaluated (without in depth consideration of how realistic this is in other resepcts) if changes in parameter values in the mechanokinetic model could completely eliminate the fast component (ΔFP_fast_) of the Pi-transient. To that end, we varied key parameter values in the model with the aim to modify the relative population of the AMD_L_ and AMD_H_ states in isometric steady-state contractions. First we noted (Fig. 6) that, for all parameter values tested, the fractional amplitude of ΔFP_slow_ decreases with increased [Pi] with corresponding increase of ΔFP_fast_, as also reflected in [Pi]-induced changes in Fig. 3b, c. Second, changes in parameter values also have marked effects on the fractional amplitudes of ΔFP_fast_ and ΔFP_slow._ As one example, an increase in x_1_ and a reduction of x_2_ lead to an increased ΔFP_slow_ component and a corresponding decrease of ΔFP_fast_. Thus, strikingly, if we reduced x_2_ from 2 to 1 nm (with only 1 out of a 9 nm total power-stroke due to the second sub-stroke) and increased x_1_ to 9 nm, ΔFP_fast_ becomes smaller than 20% of the total force change for [Pi] up to 50 mM. We now denote the standard condition defined in Tables 1, 2 as C(7.7, 2) (× 1 = 7.7 nm and x_2_ = 2 nm) whereas the altered condition is denoted C(9,1) (see Fig. 6). Under the latter condition (C(9,1)), ΔFP_fast_ is less than 10% for [Pi] < 25 mM, usually studied in Pi-transients and related experiments (Dantzig et al. 1992; Tesi et al. 2000; Stehle 2024). In summary, a mechanokinetic model with Pi-release before the power stroke (the AMD_L_-AMD_H_ transition) predicts a complex Pi-transient dominated by a slow component (< 100 s^−1^ in the present model). However, the model also predicts the existence of a fast component (> 1000 s^−1^) with an amplitude > 5–10%. This is in contrast to the experimental results which suggest a single exponential Pi-transient and similar amplitudes and [Pi]-dependence of k_tr_ and k_Pi_.

**Fig. 7:**
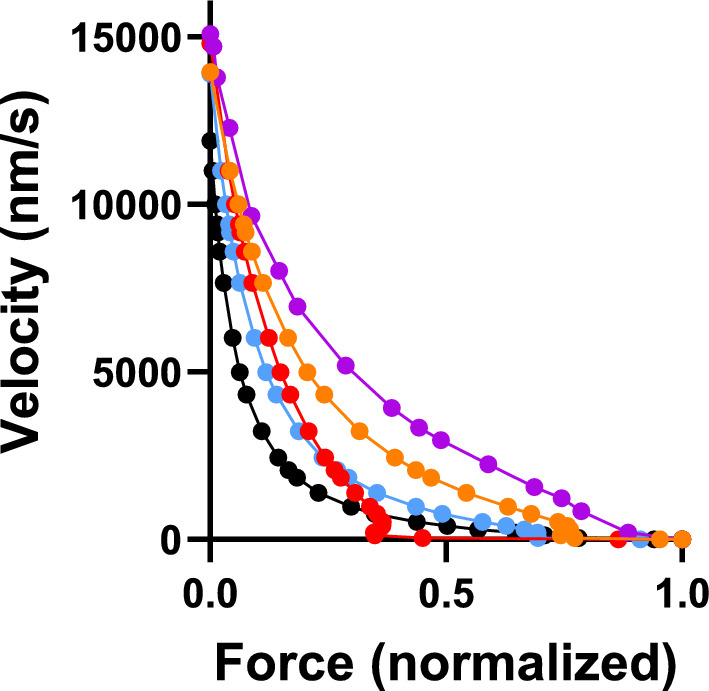
Simulated force–velocity relationships compared to experimental values for living mammalian muscle at 30 °C (purple; (Månsson et al. 1989)). Force data normalized to maximum isometric force. Simulated data based on parameter values in Tables 1, 2 (black) or as in Tables 1, 2 with modifications as follows: x_1_ = 9 nm and x_2_ = 1 nm (red), x_1_ = 9 nm and x_2_ = 1.5 nm (blue) or x_1_ = 9 nm and x_2_ = 1.5 nm and k_on_′ = 40 s^−1^ (orange)

The C(9,1)-condition gives a Pi-transient closest to a single exponential function, i.e. it has a quite small ΔFP_fast_ component. However, before evaluating this condition in greater detail, We investigated if it would reasonably well predict the force–velocity relationship of striated muscle. Unfortunately it does not (Fig. 7), giving a shape of the relationship far from the hyperbolic equation of Hill (1938). We therefore also considered a condition intermediate between C(9,1) and the standard C(7.7, 2) condition, namely (C(9,1.5) with x_1_ = 9 nm and x_2_ = 1.5 nm but otherwise the same parameter values as in Tables 1, 2. The comparision in Fig. 7 suggests that the latter choice of parameter values gives the most reasonable prediction of the force–velocity relationship, particularly if the attachment rate constant is doubled to better account for the maximum power output. We next moved on to compare the conditions C(9,1), C(9,1.5) and C(7.7,2) in different respects to better elucidate the key factors that determine the Pi-transient and initial force-redevelopment kinetics in mechanokinetic models with Pi-release before the power stroke. Despite the poor prediction of the force–velocity relationship with x_1_ = 9 nm and x_2_ = 1 nm we did not exclude this condition from additional evaluation. The reason is that it is of interest to see if any mechanokinetic model may be able to account for the Pi-transients to similar extent as the kinetic scheme in Fig. 1a. This is reasonable because the scheme in Fig. 1a cannot be at all evaluated against the force–velocity relationship.

**Fig. 8:**
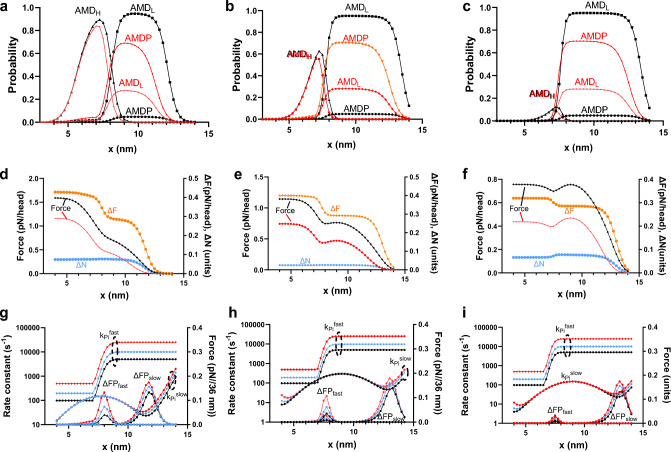
Cross-bridge distributions, force, number of attached cross-bridges and effective rate constants for Pi-induced reversal of attachment. **a**–**c** Cross-bridge distributions giving fraction of cross-bridges in biochemical states AMDP, AMD_L_ and AMD_H_ at different x-values either at 0.5 mM (black) or 25 mM Pi (red). **d**–**f** Force integrated over x (starting from x = 14 nm and progressing to x = 4 nm; left vertical axis) corresponding to cross-bridge distributions in top row for 0.5 mM (black) and 25 mM Pi (red). The difference in the integrated force (right vertical axis) between 0.5 and 25 mM Pi is depicted in orange, showing the two main components (cf. Figure 3) of the force-decrease during a Pi-transient upon change of [Pi] from 0.5 mM to 25 mM. Blue symbols depict difference in the integrated number of attached cross-bridges (right vertical axis) between 0.5 and 25 mM Pi showing that the drop in number of attached cross-bridges upon increase in [Pi] is particularly attributed to cross-bridges at x > 11 nm. **g**–**i** Rate constants (left vertical axis) and force difference (right vertical axis) between 0.5 mM Pi and high [Pi] vs x: Black, 5 mM Pi; blue, 10 mM Pi; red, 25 mM Pi. Left column **a**, **d**, **g** All parameter values as in Tables 1, 2 (C(7.7, 2)). Middle column **b**, **e**, **h** Parameter values as in left column but x_1_ = 9 nm, x_2_ = 1.5 nm (C(9, 1.5)) and k_on_′ = 40 s^−1^. Right column **c**, **f**, **i** Parameter values as in left column but x_1_ = 9 nm and x_2_ = 1 nm (C(9, 1))

The in depth analysis of the three conditions C(7.7, 2), C(9,1) and C(9,1.5) (the latter with attachment rate constant k_on_′ increased from 20 to 40 s^−1^) is depicted in Fig. 8. The analysis first clarifies the origin of the main differcnces in the fractional contribution of ΔFP_slow_ and ΔFP_fast_ (Fig. 6) based on the [Pi]-induced changes in cross-bridge distributions. Moreover, it shows that, in terms of the present mechanokinetic model, the reduction in the number of strongly attached cross-bridges with increased [Pi] is primarily due to a reduced occupation probability of the AMD_L_ and AMDP cross-bridge states (at x > 11 nm). The analysis in Fig. 8 also reveals that, in addition to [Pi]-dependent components associated with ΔFP_slow_ and ΔFP_fast_, there is a component of the total isometric force (at x < 6–7 nm) that is negligibly affected by [Pi] for all the parameter values tested (see also Figs. 3 and 6). The latter component does not contribute to the Pi-transients but it contributes to the redevelopment of isometric force.

In view of the above arguments, a mechanokinetic model is not expected to predict that k_tr_ = k_Pi_. In addition to having the [Pi] independent component, the force redevelopment would also lack a fast component related to ΔFP_fast_ that contributes to the Pi-transients. The [Pi]-independent fraction of the force redevelopment corresponds to myosin heads in the region below x = 6.5 nm (Fig. 3) and we thererfore obtained a quantitative estimate of the [Pi]-independent component of force (Fint_Pind_) as the force contribution of myosin heads in this range (for further details, see Model section). This quantitative estimate is given as a function of [Pi] for different model conditions in Fig. 6. In accordance with the definition, the [Pi]-dependence is negligible. If we neglect effects due to myosin head detachment via ADP-dissociation and ATP-binding, the rate constant of the [Pi]-independent process is given by k_on_(x) because the transition is made essentially irreversible by the high value of K_LH_ at x < 6.5 nm.

**Fig. 9:**
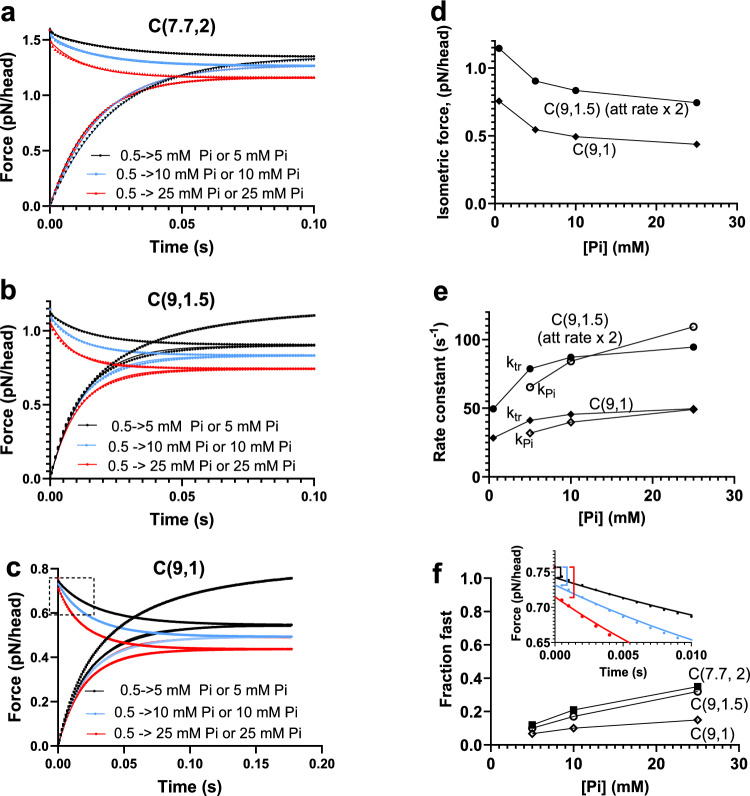
The time course of Pi-transients and force redevelopment (related to k_tr_) simulated by mechanokinetic model with different parameter values. **a** C(7.7, 2), all as in Tables 1 and 2. Best single exponential fits to the data superimposed. **b** C(9, 1.5) and k_on_′ = 40 s^−1^ otherwise as in Tables 1 and 2. Best single exponential fits to the data superimposed. Force redevelopment shown also for 0.5 mM Pi (top black). **c** C(9,1) otherwise as in Tables 1 and 2. Best single exponential fits to the data superimposed. Force redevelopment shown also for 0.5 mM Pi (top black). Area in dashed box shown in greater detail as an inset in **f**. **d** Steady-state isometric force vs [Pi] for conditions C(9,1.5) and C(9,1) from **b** and **c**. **e** Rate constants (corresponding to k_tr_ and k_Pi_) derived in single exponential fits for conditions C(9,1.5) and C(9,1) shown in **b** and **c**. **f** Fractional contribution to tension change during Pi-transient attributed to fast component for all conditions simulated in **a**–**c**. *Inset:* Early part of tension decay for the C(9,1) condition (**c**) with the very fast initial component of the tension decay indicated. Best single exponential fit as in c superimposed on the data

Taking the rate constants and force-data in Fig. 8 as starting points, we simulated Pi-transients and force redevelopment (Fig. 9) using the mechanokinetic model as described in the Model section (revolving around Eqs. 7–13). The results in Fig. 9 show that the Pi-transients, but not the force redevelopment from zero, deviate from a single exponential function with a fast initial phase. All conditions tested, C(7.7, 2) (Fig. 9a), C(9, 1.5) (Fig. 9b) and C(9,1) (Fig. 9c) predict a reduction of the isometric force with increased [Pi] that is reasonable (Coupland et al. 2001) for the preparation and temperature (25–30 °C).

This effect is summarized in Fig. 9d for the C((9, 1) and C(9,1.5) conditions, the latter with the attachment rate constant doubled (k_on_´= 40 s^-1^). It is also shown for these conditions in Fig. 9e that the effects on isometric force are coupled to reciprocal effects of increased [Pi] on k_tr_ and k_Pi_. The coupling between the change in isometric force and k_tr_ does not have exactly the same functional relationship as in a recent experimental study of guinea pig cardiac myofibrils at 10 °C (Stehle 2024). In the latter study, the force shows a clear saturation behaviour at high [Pi] whereas the dependence of k_tr_ on [Pi] is nearly linear. In our model both k_tr_ and isometric force show similar saturation behaviour. We do not investigate the basis for this difference. However, we note that the [Pi]-effects vary greatly with temperature (e.g. (Coupland et al. 2001)). Moreover, other studies using fast rabbit skeletal muscle myofibrils (Tesi et al. 2000) have indicated more similar saturation behaviour between the variation with [Pi] of k_tr_ and isometric force.

Recent work (Moretto et al. 2022) suggests that the assumption of multistep Pi-binding would mitigate the differences from experimental results of the mechanokinetic model by removing the fast phase of tension decay from the Pi-transients following increases in [Pi].

**Fig. 10:**
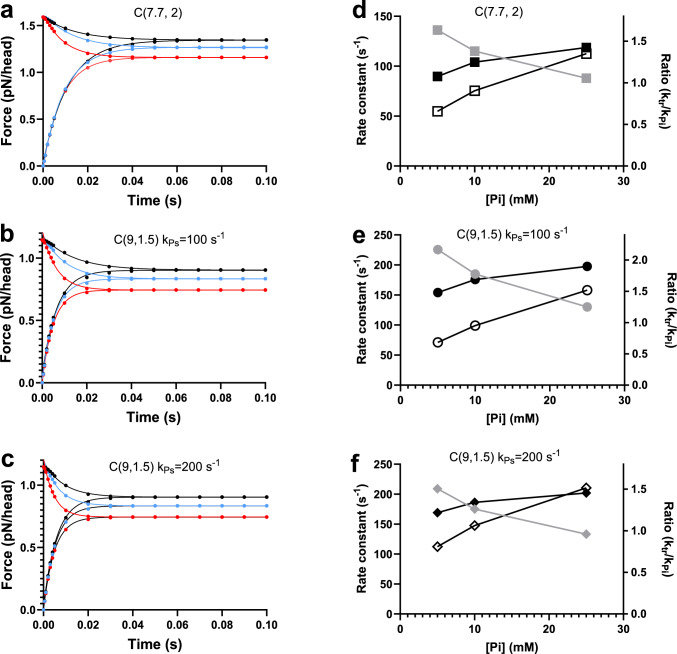
Simulation of Pi-transients and rate of force redevelopment vs [Pi] for simplified representation of multistep (two-step) Pi-release. **a** Time courses with contribution of top (slow) and bottom (fast) scheme in Fig. 4 with respective amplitudes determined from the mechanokinetic model for C(7.7, 2) condition as described in the Model section. Pi-transients from 0.5 mM to higher final [Pi]. Final Pi-concentration either 5 mM (black), 10 mM (blue) or 25 mM (red). Single exponential fits superimposed on the data. **b** Simulations using mechanokinetic model as in a but for the C(9, 1.5) condition with the Pi-release rate (k_Ps_) equal to k_on_, i.e. k_ps_ = k_on_ = 100 s^−1^. Single exponential fits superimposed on the data. **c** Simulations using mechanokinetic model as in a but for the C(9, 1.5) condition with k_Ps_ = 200 s^−1^ and k_on_ = 100 s^−1^. Single exponential fits superimposed on the data. **d** Estimates of k_Pi_ (open symbols) and k_tr_ (filled symbols) from single exponential fits and their ratio for the condition in **a**. **e** Estimates of k_Pi_ (open symbols) and k_tr_ (filled symbols) from single exponential fits and their ratio for the condition in **b**. **f** Estimates of k_Pi_ (open symbols) and k_tr_ (filled symbols) from single exponential fits and their ratio for the condition in **c**

We tested this idea by a simplified treatment described in the Methods and in Fig. 4. For the Pi-transients we obtained the amplitude of the fast (lower scheme in Fig. 4) and slow (upper scheme in Fig. 4) component from ΔFP_fast_ and ΔFP_slow_, respectively where the latter values were obtained from the mechanokinetic modelling. The amplitudes for redevelopment of force from zero, on the other hand, were obtained from the integral force at the given [Pi] below (fast component; lower scheme) and above (slow component; upper scheme) the x-value of the force-integral′s inflection point. We focused the analysis on the conditions C(7.7, 2) and C(9,1.5) that give most faithful reproduction of the force–velocity relationship. The results are depicted in Fig. 10. As expected, both the Pi-transients and the force redevelopment are well approximated by single exponentials. However, k_tr_ is consistently higher than k_Pi_, particularly at low [Pi]. Whereas this difference can be reduced by altered parameter values i, e.g. higher ratio, k_Ps_/k_on_ (Fig. 10e vs 10f) it seems difficult to completely eliminate the differences. The faster average rates compared to Fig. 9 are due to choice of rate constants k_on_ and k_on-_ as for the analysis using kinetic schemes (Fig. 5). These values are faster than the corresponding rates in the mechanokinetic model from which we, however, take the amplitudes of the fast and slow component (details in Methods) corresponding to the relative contribution of the two parallel schemes in Fig. 4.

## Discussion

### Simple kinetic schemes and experimental complexities

We first corroborated previous findings that the single exponential Pi-transients and the similar values of k_tr_ and k_Pi_ at a given [Pi] is accounted for by a kinetic scheme where Pi-release occurs after force-generation. However, we also show (which is not explicitly clear from previous work) that, for its success in these regards (without additional assumptions (Caremani et al. 2008); see below), force-generation must be conincident with the rate limiting actin attachment step or, alternatively, the transition from a weak-binding to a strong-binding actomyosin state. A separate force-generating step of the (Huxley and Simmons 1971) type (a power stroke), subsequent to the rate-limiting step would not work because it predicts a biphasic Pi-transient and different apparent k_Pi_ and k_tr_ as in the schemes in Fig. 1b and c. Our interpretation of previous results (e.g. (Dantzig et al. 1992; Tesi et al. 2000, 2002) in relation to the Scheme in Fig. 1a differs from that of (Stehle 2024) using simulations based on several different kinetic schemes to evaluate effects of varied [Pi] on k_tr_ and isometric force. Stehle (Stehle 2024) interpreted the previous uses of our scheme Fig. 1a on the assumption that it includes a force-generating transition separate from the rate limiting attachment step, i.e. as our scheme in Fig. 1b. However, to our understanding, whereas the existence of a separate force-generating transition was implicity assumed by several authors (Kawai and Halvorson 1991; Dantzig et al. 1992; Tesi et al. 2000, 2002; Ranatunga et al. 2002; Kawai et al. 2021) in the previous work, it was not implemented in the computations. Thus, the consequences, e.g. biphasic Pi-transients, were not explicitly exposed. We therefore equate our Fig. 1a to the scheme “(FR)P” in the terminology of (Stehle 2024) in which force-generation is coincident with the rate-limiting attachment step and followed by fast Pi-release. Further, we equate our scheme in Fig. 1b to the scheme “RFP” of (Stehle 2024). In the latter scheme,the rate-limiting attachment step comes first, followed by a separate fast force-generating event and fast Pi-release. Finally, we equate our scheme in Fig. 1c with the “RPF” scheme of (Stehle 2024). In this scheme a separate rate-limiting attachment is followed by fast Pi-release and fast force-generation in that order. It is of interest to note that our scheme in Fig. 1a (the scheme fitting best to both the Pi-transient and force-redevelopment data), would also give a faithful prediction of the coupling between [Pi] effects on force and k_tr_ studied by (Stehle 2024). Importantly we did not consider any kinetic schemes (or mechanokinetic model) with slow Pi-release or slow force-generation, possibly with Pi-release being rate-limiting for the entire cycle. Such a step would necessarily occur following strong actin-binding (e.g. see review of (Robert-Paganin et al. 2020) and would therefore also rate-limit the maximum velocity of shortening in addition to the actin-activated ATP turnover. A slow transition in actin-attached states may occur in the presence of drugs (Rahman et al. 2018; Rassier and Månsson 2025) and thereby shift the rate-limiting steps for both actin-activated ATPase and maximum shortening velocity as well as their relationships to Pi-release. However, physiologically, the maximum shortening velocity is known to be limited by the ADP-release rate (Siemankowski et al. 1985) and ATP induced cross-bridge detachment (Nyitrai et al. 2006).

We did not further consider the coupling between effects of varied [Pi] on force and k_tr_ (Stehle 2024) using the mechanokinetic model. The reason is that it requires a consistent set of experimental data for comparing force and k_tr_ obtained in the same preparations. Moreover, we cannot easily use the experimental data of (Stehle 2024) to analyse the phenomenon because our model parameter values are for fast skeletal muscle at 25–30° whereas the data of (Stehle 2024) are for guinea pig cardiac muscle at 10 °C.

The mentioned differences in experimental preparations and temperature, highlight a general challenge when interpreting any experimental results in terms of models. It is critically important that the model parameter values are relevant for interpreting a given set of experimental results. In this regard there are more complications than differences in species, preparation and temperature. One such complication is the non-uniform sarcomere behaviour that was observed in cardiac myofibrils (Stehle 2017) upon increase but not decrease in [Pi]. Such effects could explain why the k_Pi_ measured in Pi-transients due to increased [Pi] had been generally found to be appreciably higher than k_tr_ at similar [Pi] (Millar and Homsher 1990; Tesi et al. 2000). On the other hand, Pi-transients due to lowered [Pi], possible to study in myofibrils, showed k_tr_ ≈ k_tr_ (Tesi et al. 2000; Stehle 2017). The results from myofibrils raise the possibility that all experiments with increase in [Pi] also in muscle cells suffer from similar complications. However, one cannot exclude differences between cardiac myofibrils and the more often used skinned fast skeletal muscle cells or skeletal muscle myofibrils. Interestingly, in this connection, Kaya (Kaya 2025) recently (see also (Hwang et al. 2021)) presented data suggesting key differences in Pi-effects on cardiac and skeletal muscle myosin. However, similar emerging sarcomere non-uniformities as those speeding up the tension drop after a sudden increase in [Pi] in cardiac myofibrils (Stehle 2017) have been found to speed up relaxation from isometric contraction following reduced myofibrillar [Ca^2+^] in both living skeletal muscle cells (Edman and Flitney 1982) and skeletal muscle myofibrils (Poggesi et al. 2005). These findings lend support to the idea that the Pi-transients upon increased [Pi] are speeded up due to sarcomere-non-uniformities independent of muscle preparation. Accordingly, we tentatively interpret the experimental evidence to mean that k_Pi_ ≈ k_tr_.

The experimental evidence for single exponential Pi-transients as well as single exponential force redevelopment at varied [Pi] are somewhat surprising considering complexities in the experiments. In addition to the abovementioned sarcomere non-uniformity, the continuous changes in isometric force in both Pi-transients and during force redevelopment would be associated with changes in force in the thick filaments, in turn expected to modify mechanosensing based thick filament activation level (Linari et al. 2015; Brunello et al. 2023) (reviewed in (Brunello and Fusi 2024)). Nevertheless, good approximations with single exponentials have been reported for the Pi-transients and force redevelopment in previous experiments using both skeletal muscle (Brenner and Eisenberg 1986; Dantzig et al. 1992) and cardiac (Stehle 2017) myofibrils. The mechanosensing effect has been incorporated into mechanokinetic models (Mijailovich et al. 2021) and the possibility to assess effects of varied Pi (including Pi-transients and force redevelopment) using such models would be of interest in future work. Other effects to possibly incorporate in future modelling efforts are those due to sarcomere-length changes that occur because of various forms of series compliance during changes in tension. This includes compliance in myofilaments, other structures in the muscle (or myofibril) and in the attachment of the preparation to the mechanical apparatus. The effects of compliant elements on the time course of tension change can be quite substantial as indicated by effects of eliminating the external compliance by clamping the sarcomere-length during the rise of force. This is exemplifed both by effects on the rate of redevelopment of force of a skinned rabbit psoas muscle fibre after a shortening-restretch protocol (Brenner and Eisenberg 1986) and the rise of force in a twitch of a cardiac trabecula (e.g. (Caremani et al. 2016)). Mechanical studies of Pi-transients and force redevelopment in myofibrils and muscle cells ususally do not apply sarcomere length control as in the mentioned studies. Whereas the effects can partly be addressed by modelling, more experiments would be of value to finally verify the detailed characteristics of both Pi-transients and force redevelopment. This includes final corroboration of the results suggesting that k_tr_ ≈ k_Pi_ at varied [Pi] both for up-jumps and down-jumps in [Pi]. It also includes further tests of the notion that both the Pi-transients and the force redevelopment follow nearly single exponential time courses. Finally, it is of interest to further investigate possible differences between cardiac and skeletal muscle myofibrils (Kaya 2025).

Fortunately, in view of the complexities associated with large scale perturbations (particularly force redevelopment experiments), effects of varied [Pi] on key rate constants for the actin-myosin interaction seem to be corroborated by effects of [Pi] in small scale perturbation experiments (reviewed in (Kawai 2025)). Such perturbations, that do not involve large changes in sarcomere length, include analysis of the tension response to low-amplitude sinusoidal length oscillations applied to muscle fibres (Kawai and Halvorson 1991), pressure jumps (Fortune et al. 1991) and temperature jumps (Ranatunga 1999). Interestingly, the effects of varied [Pi] on the results of these perturbations have generally been found to be well accounted for by a kinetic scheme similar to that in Fig. 1a (reviewed in (Kawai 2025)). That is, they are accounted for by a scheme that also accounts well for the Pi-transients and force redevelopment (ignoring limitations in other regards as considered here and elsewhere (Linari et al. 2010)). Due to uncertainties in the underlying experiments as discussed in detail above, we did not aim for quantitatively perfect fits to experimental data and in some instances (e.g. for the schemes) we use parameter values from experiments with increasing Pi that do not consider possible problems with sarcomere non-uniformities. This is justified to illustrate key principles.

As pointed out above, the only tested simple kinetic scheme that can account for both the Pi-transient and force redevelopment data, is that in Fig. 1a with a main characteristic that force-generation is conincident with cross-bridge attachment. It is particularly notable that the scheme with a transient actin-attached state before force-generation would not be consistent with a single exponential Pi-transient, even if force-generation occurs prior to Pi-release (Fig. 1b). The idea that force is present immediately upon attachment is a characteristic of the early mechanokinetic model of Huxley (Huxley 1957). In the latter model the force-development is attributed to thermal fluctuations that strain the cross-bridge to its maximum force already before attachment. The problem with such a model was, however, realized already by (Huxley and Simmons 1971). Particularly, such a mechanism with its core dependence on thermal fluctuations, would account neither for the high isometric force nor the high power-output of muscle. Based on the additional evidence provided by the tension transients in response to fast length steps, Huxley and Simmons (Huxley and Simmons 1971) therefore suggested that force is developed step-wise, *after* cross-bridge attachment to actin. Later, similar ideas have gained support from a bulk of structural (Rayment et al. 1993; Klebl et al. 2025), single molecule mechanics (Woody et al. 2019) and biochemical evidence (reviewed in (Robert-Paganin et al. 2020; Rassier and Månsson 2025)) and the different states have been associated with appropriate biochemical intermediates and coarse-grain structure. This has led to mechanokinetic models integrating a swinging lever-arm as the main force-, and motion-generating structural change. An obvious problem with the treatment using kinetic schemes similar to that in Fig. 1a which accounts well for the Pi-transients (see also references above) is that a power stroke similar to that originally suggested by (Huxley and Simmons 1971), does not seem to have a role. Attempts to integrate such transitions in the schemes in Fig. 1b and c clearly fail by leading to different apparent k_tr_ and k_Pi_ values and a double exponential Pi-transient. Moreover, the schemes in Fig. 1b and c do not account for the coupling between [Pi]-effects on force and k_tr_ (Stehle 2024). The mentioned results seem to pose a severe limitation either to the role of the power stroke or to the use of kinetic schemes to interpret the Pi-effects. A possible way around the problem was proposed by Caremani et al. (Caremani et al. 2008) who suggested that a force-generating power stroke occurs in all attached cross-bridge states, i.e. in both the biochemical states with and without Pi at the active site in Fig. 1a. This means that either of these states should be subdivided into one pre-power-stroke and one post-power-stroke state in rapid equilibrium with each other, i.e. with the biochemical transition of Pi-release orthogonal to the mechanical power stroke transition. However, it was also pointed out (Caremani et al. 2008) that the success of such a model relies on the assumption that the equilibrium constant for the power stroke is the same with and without Pi at the active site. In view of this uncertainty and the likely existence of a range of cross-bridge strains, it is important to also test the alternative approach to resolve the problems using a more complex mechanokinetic model as indicated previously (Smith 2014).

### Mechanokinetic model

Our mechanokinetic model is rather simple with four strongly attached states (each existing at a range of x-values; strain-levels). The model does not include recently proposed ideas such as branched pathways (Linari et al. 2010; Debold et al. 2011, 2013; Caremani et al. 2013; Jarvis et al. 2018; Mijailovich et al. 2021; Scott et al. 2021; Marang et al. 2025), loose coupling between Pi-release and the power-stroke (Malnasi-Csizmadia and Kovacs 2010; Caremani et al. 2013, 2015) or multistep Pi-release (Llinas et al. 2015; Planelles-Herrero et al. 2017; Robert-Paganin et al. 2020) found to improve the reproduction of several experimental findings in addition to having a fair degree of experimental support (Moretto et al. 2022). Our main finding from use of the mechanokinetic model is that consideration of varying strains of attached cross-bridges would be expected to lead to complex time courses of the Pi-transients that would also show different kinetics than the force redevelopment from zero. For some sets of parameter values the latter difference is small. Thus, we found that > 90% of the Pi-transient can be described by a single exponential with similar rate constant as the force redevelopment with only a small fraction being due to a fast component. Interestingly, using a mechanokinetic model rather similar to that used here (Smith 2014), with a fast Pi-release before a power stroke albeit with somewhat different model structure in other regards and different values of the model parameters, (Smith 2014), found that the Pi-transients were well described by single exponential functions with k_tr_ ≈ k_Pi_. He explained this, as is the case for our dominant slow component of the Pi-transient as being due to detachment (reversal of attachment) from the AMD_L_ via the AMDP to the MDP state (using our terminology for the states). We conclude that a mechanokinetic model gives different predictions than a seemingly analogous kinetic scheme. However, using our model with varied parameter values we could not fully account for both Pi-transients and the force redevelopment without additional assumptions. The previous findings of (Smith 2014), however, suggests that it may be possible to obtain a better fit by further changes in parameter values and, possibly, minor changes in model structure.

### Higher order effects – multistep Pi-release

We showed previously that a multistep Pi-release model leads to single exponential Pi-transients, following a brief lag, when approximated using a simplified kinetic scheme (Moretto et al. 2022). However, we did not consider whether k_tr_ and k_Pi_ could be made reasonably similar in such a model. The present analysis corroborates the prediction of single exponential behaviour with a lag also using our schemes in Fig. 4 developed from the mechanokinetic model. However, the analysis predicts different values and [Pi]-dependence of k_tr_ and k_Pi_ with reduced difference for an increased k_Ps_/k_on_ ratio. It is important to note in this connection that too high value of k_Ps_ would be inconsistent with slow appearance of Pi in solution, an important feature of the multistep model. Possibly this issue may be accounted for by poorly understood properties of the Pi-release, e.g. different mechanisms of Pi-release and Pi-rebinding for cross-bridges in AMD_L_ state at high x than in AMD_H_ cross-bridges at x ≈ x_1_. For instance, if there is multistep Pi-release only in the latter case, the fast phase of the Pi-transient would be eliminated without any effects on the slow dominant phase that is the primary determinant of both of k_tr_ and k_Pi_. However, one must also remember that our simulations of the multistep model treats the process in a greatly simplified way with only one strain level each (rather than a distribution) representing the fast and slow component. It is important in future studies to more generally characterize the kinetics of Pi-binding and release associated with Pi-binding sites on myosin outside the active site. Finally, it will also be important with additional studies to conclusively show that k_Pi_≈k_tr_ in experiments under all conditions, i.e. not only in cardiac myofibrils, as there are uncertainties in that regard (cf. Tesi et al. 2000; Stehle 2017)).

## Conclusions

The present study corroborates previous results that for a simple kinetic scheme to account for Pi-transients and their [Pi] dependence, force-generation must be effectively coincident with cross-bridge attachment, thereby also occurring before Pi-release from the active site. As we argue above, the idea of force-generation coincident with cross-bridge attachment is not straightforwardly consistent with other findings including the high power-output of muscle and the idea of a separate force-generating power stroke. Whereas a potential modification of kinetic schemes has been proposed (Caremani et al. 2008) to circumvent the problem, it is important to also test a mechanokinetic model for this purpose because it takes into account an expected range of cross-bridge strains in a real muscle. Specifically, we tested a model with Pi-release before force-generation. We found that such a model would lead to Pi-transients and force redevelopment at varied [Pi] that are seemingly quite different compared to these phenomena predicted by analogous kinetic schemes. Generally, the predictions of the mechanokinetic model would be rather complex. First, the Pi-transient would be a double exponential with two Pi-dependent components, one very fast (> 1000 s^−1^) and one rather slow (< 100 s^−1^). Second, associated with the existence of a fast component in the Pi-transient, k_tr_ for the single-exponential force redevelopment would be similar only to the rate constant of the slow component of the Pi-transient. However, despite the observed complexity, it is of interest to note that we could find parameter values that predict Pi-transients with quite similar rates as for the force redevelopment with only a small fast component. Therefore (see also (Smith 2014) for similar results), it cannot be excluded that a set of parameter values does exist to allow mechanokinetic models with Pi-release before the power-stroke to fully account for Pi-transients and force development at varied [Pi], without additional assumptions. Otherwise, we showed that a combination of a simplified approximation of our mechanokinetic model with a multistep Pi-release mechanism would give a more faithful reproduction both of Pi-transients and the force redevelopment. However, further studies are required to finally clarify the issue. It will also be important in the future with more experiments to clarify any differences between muscle types and to corroborate single exponential Pi-transients and force redevelopment with k_tr_ ≈ k_Pi_.

## Data Availability

The data that support the findings of this study are available from the corresponding author upon reasonable request.
